# Comparative effectiveness and safety of almonertinib in EGFR-mutated non-small cell lung cancer: an integrated evidence synthesis

**DOI:** 10.3389/fphar.2026.1932051

**Published:** 2026-10-09

**Authors:** Biaoxue Rong, Hui Pan, Tian Fu, Yanfeng Liu, Pan Yang, Jiaying Zhang, Ying Wang

**Affiliations:** 1 Department of Oncology, The First Affiliated Hospital of Xi’an Medical University, Xi’an, China; 2 Department of Respiratory Medicine, Gansu Provincial Hospital, Lanzhou, China; 3 Department of Respiratory Medicine, Jining First People’s Hospital, Jining, China

**Keywords:** almonertinib, comparative effectiveness, EGFR mutation, meta-analysis, non-small cell lung cancer, tyrosine kinase inhibitor

## Abstract

**Background:**

Comparative evidence regarding the efficacy and safety of almonertinib in epidermal growth factor receptor (EGFR)-mutated non-small cell lung cancer (NSCLC) remains fragmented across randomized and real-world studies. We conducted a systematic review and meta-analysis to comprehensively evaluate the comparative clinical performance of almonertinib.

**Methods:**

Eight electronic databases were systematically searched from inception to March 2025. Comparative studies evaluating almonertinib versus active control regimens in patients with EGFR-mutated NSCLC were included. Random-effects meta-analyses were performed to synthesize objective response rate (ORR), disease control rate (DCR), progression-free survival (PFS), overall survival (OS), and treatment-related adverse events. Methodological quality was assessed using the Cochrane Risk of Bias tool and analogous domains for observational studies, and certainty of evidence was evaluated using the GRADE framework.

**Results:**

Fifteen comparative studies involving 1,902 patients met the eligibility criteria. Compared with control regimens, almonertinib was associated with higher pooled ORR (RR = 1.33, 95% CI 1.08–1.65) and DCR (RR = 1.14, 95% CI 1.03–1.26). However, after trim-and-fill adjustment for potential publication bias, the 95% CIs for both ORR and DCR included the null value, suggesting that the observed response benefits may be influenced by small-study effects. No statistically significant differences were observed for pooled PFS or OS. Almonertinib demonstrated a generally favorable safety profile, with a lower incidence of diarrhea, while rates of grade ≥3 adverse events and other major toxicities were broadly comparable between groups. Integrated analyses further suggested consistent concordance between ORR and DCR across most studies, supporting improved short-term disease control despite moderate-to-substantial between-study heterogeneity.

**Conclusion:**

Current comparative evidence suggests that almonertinib may improve tumor response and disease control while maintaining a favorable tolerability profile in patients with EGFR-mutated NSCLC. Although favorable objective response outcomes were observed, these benefits may be influenced by small-study effects, and definitive survival advantages remain uncertain. These findings support almonertinib as a clinically meaningful therapeutic option, while underscoring the need for cautious interpretation and for larger, high-quality comparative trials with longer follow-up to further define its long-term clinical value.

**Systematic Review Registration:**

https://doi.org/10.17605/OSF.IO/ZDF2N; Identifier: OSF.IO/ZDF2N.

## Introduction

Lung cancer remains the leading cause of cancer-related mortality worldwide. According to GLOBOCAN 2022 estimates, approximately 2.48 million new cases of lung cancer were diagnosed globally, accounting for 12.4% of all incident cancers, while an estimated 1.82 million deaths were attributable to lung cancer, representing 18.7% of all cancer deaths (Bray et al., 2024). Non–small-cell lung cancer (NSCLC) constitutes the predominant histological subtype, and molecular profiling has transformed its therapeutic landscape. Among actionable alterations, activating mutations in the epidermal growth factor receptor (EGFR) gene are among the most clinically relevant, occurring in roughly 10%–15% of NSCLC in Western populations and at substantially higher frequencies in East Asian patients (Bouchard and Daaboul, 2025). The two most common sensitizing variants—exon 19 deletions and the L858R point mutation in exon 21— confer sensitivity to EGFR tyrosine kinase inhibitors (EGFR-TKIs) and have become standard biomarkers guiding first-line targeted therapy (Wang et al., 2024). The development of EGFR-TKIs has progressed through successive generations. First-generation agents (e.g., gefitinib and erlotinib) and second-generation inhibitors (e.g., afatinib and dacomitinib) improved outcomes relative to chemotherapy in EGFR-mutated NSCLC; however, acquired resistance inevitably emerges, most frequently through the gatekeeper T790M mutation in exon 20 (Wang et al., 2024; Westover et al., 2018). Third-generation EGFR-TKIs were engineered to inhibit both sensitizing and T790M mutations while sparing wild-type EGFR, thereby reducing class-associated toxicities such as rash and diarrhea. Osimertinib, the first widely adopted third-generation agent, demonstrated superior efficacy versus first-generation TKIs in the FLAURA trial and is now a global standard of care (Cross D. A. et al., 2014; Cheng et al., 2021). Almonertinib (also known as aumolertinib or HS-10296) is an orally administered, irreversible third-generation EGFR-TKI developed in China. Preclinical and early clinical data indicated potent activity against common sensitizing mutations and the T790M resistance mutation, together with a favorable safety profile characterized by lower rates of dermatologic and gastrointestinal adverse events than earlier-generation inhibitors (Shirley and Keam, 2022).

The pivotal phase III AENEAS trial established the registrational evidence base for almonertinib in untreated EGFR-mutated advanced NSCLC. In that study, almonertinib significantly prolonged progression-free survival (PFS) compared with first-generation gefitinib (median PFS 19.3 versus 9.9 months; hazard ratio [HR] 0.46; 95% confidence interval [CI] 0.36–0.60; P < 0.001), with a manageable toxicity profile (Lu et al., 2022). These findings supported approval of almonertinib for first-line treatment in China and have prompted growing interest in its comparative effectiveness relative to other EGFR-directed strategies. Nevertheless, AENEAS alone cannot address the full spectrum of clinical questions facing practitioners. Real-world cohorts, head-to-head comparisons with other third-generation agents, evaluations in special populations (including elderly patients and those with brain metastases), and studies reporting patient-centered endpoints beyond PFS continue to accumulate, particularly within China where almonertinib has been widely adopted since regulatory approval (Li et al., 2025). Moreover, evidence is dispersed across randomized controlled trials (RCTs) and nonrandomized comparative studies, often with heterogeneous comparators—first-generation EGFR-TKIs, other third-generation agents, or chemotherapy—and with variable outcome definitions, imaging assessment schedules, and follow-up durations (Mok et al., 2017).

Systematic synthesis of this expanding literature is therefore warranted. Although narrative reviews and single-trial analyses provide useful context, they cannot quantitatively integrate effect estimates or comprehensively appraise consistency across designs and settings. Meta-analysis offers a structured framework to pool comparable outcomes, explore between-study heterogeneity, and grade the certainty of evidence—an approach increasingly expected by international guideline developers and journal readers (Parums, 2021). To date, no comprehensive quantitative synthesis has integrated the available comparative clinical evidence on almonertinib spanning both efficacy and safety endpoints across diverse study designs. Accordingly, we conducted a systematic review and meta-analysis of comparative clinical studies evaluating almonertinib in patients with EGFR-mutated NSCLC. We searched major bibliographic databases and regional literature sources without language restriction and included studies that compared almonertinib with an active or standard control and reported at least one prespecified efficacy or safety outcome. Fifteen eligible studies were identified, comprising three English-language publications and twelve Chinese-language reports published between 2022 and 2025, including the landmark AENEAS RCT and a range of observational and comparative cohort investigations conducted in tertiary oncology centers. Primary efficacy outcomes of interest included objective response rate (ORR), disease control rate (DCR), PFS, and overall survival (OS), where mature data were available; safety outcomes encompassed treatment-related adverse events of special interest, including rash, diarrhea, elevated aminotransferases, and QT-interval prolongation. Study quality was assessed using the Cochrane Risk of Bias tool for randomized trials; for nonrandomized studies, analogous Cochrane RoB domains were applied where applicable, and the certainty of evidence was evaluated with the GRADE framework.

The primary aim of this meta-analysis was to provide an integrated estimate of almonertinib’s comparative effectiveness and tolerability in EGFR-mutated NSCLC. Secondary objectives included examining consistency of findings across study designs, exploring potential sources of heterogeneity, and identifying gaps in the current evidence base to inform clinical decision-making and future research. By consolidating data from RCTs and comparative observational studies, we sought to deliver a balanced, internationally readable synthesis that complements registrational trial evidence and supports evidence-based use of almonertinib in contemporary thoracic oncology practice.

## Methods

### Study registration and search strategy

The protocol for this systematic review and meta-analysis was registered on the Open Science Framework (OSF) (https://doi.org/10.17605/OSF.IO/ZDF2N). The review was conducted and reported in accordance with the PRISMA 2020 guidelines. A computerized systematic search was performed across eight electronic databases, comprising four international sources (PubMed, Embase, the Cochrane Library, and Web of Science) and four Chinese-language sources (the Chinese Biomedical Literature Database [CBM], China National Knowledge Infrastructure [CNKI], the Chinese Science and Technology Journal Database [VIP], and Wanfang Data). Each database was searched from inception to March 2025, without language restriction. The search strategy combined subject headings with free-text terms, connected using Boolean operators and adapted to the syntax of each database. English search terms included “almonertinib,” “aumolertinib,” “HS-10296,” “non-small cell lung cancer,” and “NSCLC,” combined with terms such as “EGFR,” “tyrosine kinase inhibitor,” “randomized controlled trial,” and “comparative study.” Reference lists of eligible studies and relevant reviews were manually screened to identify additional potentially eligible studies.

### Eligibility criteria

Studies were eligible if they (1) enrolled patients with EGFR-mutated NSCLC; (2) compared almonertinib or an almonertinib-containing regimen against an active comparator, including other EGFR-TKIs, chemotherapy, or combination regimens; (3) reported at least one prespecified efficacy outcome (ORR, DCR, PFS, or OS) or safety outcome; and (4) were randomized controlled trials or comparative observational studies with a concurrent control group. Reviews, case reports, single-arm studies, dissertations, conference abstracts without extractable data, and studies with incomplete outcome or baseline data were excluded.

Across the included studies, NSCLC diagnosis was based on histological and/or cytological confirmation as reported in the original articles. Several studies additionally cited national clinical guidelines or referenced diagnostic criteria, whereas a formal WHO histological classification edition was rarely specified. EGFR mutation positivity was required in all studies.

### Outcome measures

Efficacy outcomes were defined according to RECIST version 1.1. Objective response rate (ORR) was defined as the proportion of patients achieving a complete response (CR) or partial response (PR); disease control rate (DCR) was defined as the proportion achieving CR, PR, or stable disease (SD). Progression-free survival (PFS) was defined as the time from treatment initiation to disease progression or death from any cause, and overall survival (OS) as the time from treatment initiation to death from any cause. Safety outcomes comprised treatment-related adverse events graded according to the Common Terminology Criteria for Adverse Events (CTCAE) or the study-defined equivalent, including the overall adverse drug reaction (ADR) rate, the incidence of grade ≥3 ADRs, and prespecified adverse events of interest (diarrhea, rash, nausea/vomiting, bone marrow toxicity, and hepatic or renal toxicity).

### Study selection

Search results were imported into reference management software, and duplicates were removed. Two investigators independently screened titles and abstracts against the eligibility criteria, followed by full-text assessment of potentially eligible articles. Discrepancies at either stage were resolved through discussion or, when necessary, adjudication by the corresponding author. The study selection process was conducted in accordance with the PRISMA 2020 guidelines, and the corresponding flow of records is reported in the Results (Figure 1).

**FIGURE 1:**
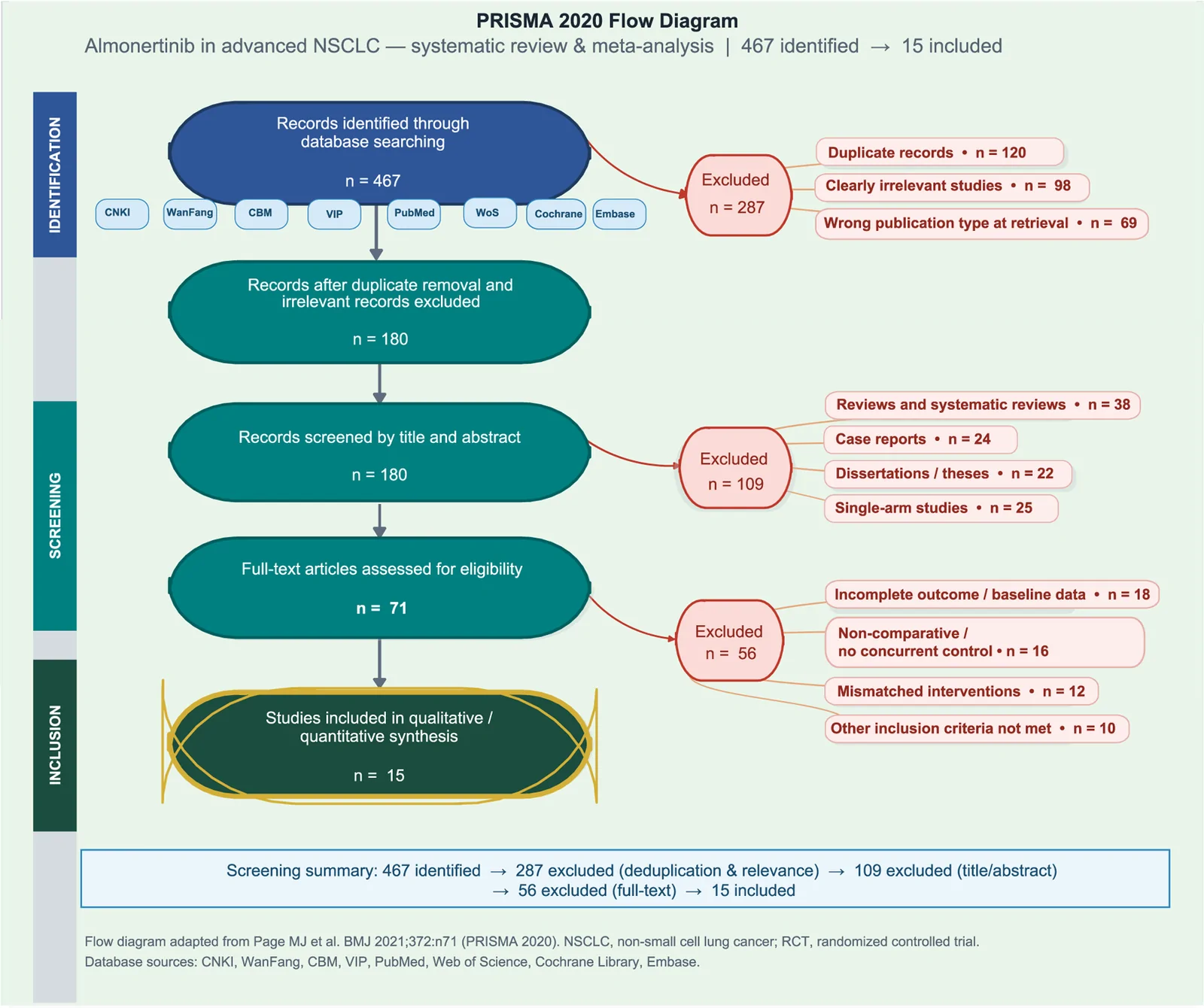
PRISMA 2020 flow diagram illustrating the study selection process for the systematic review and meta analysis. A total of 467 records were identified through systematic searches of CNKI, WanFang, CBM, VIP, PubMed, Web of Science, Cochrane Library, and Embase. After removal of duplicate records, clearly irrelevant studies, and records with inappropriate publication types, 180 records remained for title and abstract screening. Following screening, 71 full text articles were assessed for eligibility. Fifty-six articles were subsequently excluded because of incomplete outcome or baseline data, non-comparative or single-group designs without a concurrent control arm, mismatched interventions, or failure to meet the predefined eligibility criteria. Comparative observational studies with concurrent controls were eligible for inclusion. Ultimately, 15 comparative studies were included in the qualitative and quantitative syntheses. Abbreviations: CBM, Chinese Biomedical Literature Database; CNKI, China National Knowledge Infrastructure; Embase, Excerpta Medica Database; n, number of records, articles, or studies; NSCLC, non-small cell lung cancer; PRISMA, Preferred Reporting Items for Systematic Reviews and Meta-Analyses; RCT, randomized controlled trial; VIP, Chinese Science and Technology Journal Database; WoS, Web of Science. Flow diagram adapted from Page MJ, McKenzie JE, Bossuyt PM, et al. The PRISMA 2020 statement. BMJ. 2021;372:n71.

### Data extraction

Two investigators independently extracted data using a standardized, pre-piloted extraction form, including study identification, design, sample size, patient baseline characteristics, treatment regimens, and efficacy and safety outcomes. Discrepancies were resolved through discussion or adjudication by the corresponding author. When outcome data were not directly reported, values were derived from available numerators and denominators (e.g., ORR or DCR calculated from CR/PR/SD/PD counts), as specified in the relevant table footnotes.

### Quality assessment

Methodological quality was independently assessed by two reviewers using the Cochrane Risk of Bias tool for randomized controlled trials. For nonrandomized comparative studies, analogous Cochrane RoB domains were applied where applicable, with non-applicable domains marked as NA. The certainty of evidence for each outcome was subsequently graded using the GRADE framework (High, Moderate, Low, or Very low), with downgrading considered for risk of bias, inconsistency, indirectness, imprecision, and publication bias.

### Statistical analysis

Dichotomous outcomes were pooled as risk ratios (RRs) with 95% confidence intervals (CIs) using the Mantel–Haenszel method; time-to-event outcomes were pooled as hazard ratios (HRs) with 95% CIs. A random-effects model (DerSimonian–Laird) was applied *a priori* given anticipated heterogeneity across study designs. Heterogeneity was quantified using the I^2^ statistic and Cochran’s Q test. A continuity correction of 0.5 was applied only to studies with zero events in one arm. Prespecified subgroup analyses were performed according to comparator regimen. Leave-one-out sensitivity analyses assessed the robustness of pooled estimates to sequential exclusion of individual studies. Publication bias for ORR and DCR was evaluated using Egger’s regression test, Begg’s rank correlation test, and the trim-and-fill method. All analyses were performed using the meta package (version 8.5) in R software, with two-sided P < 0.05 considered statistically significant unless otherwise specified.

## Results

### Study selection

A total of 467 records were identified through searches of eight electronic databases, including CNKI, WanFang, CBM, VIP, PubMed, Web of Science, Cochrane Library, and Embase (Figure 1). After removal of 120 duplicate records, exclusion of 98 clearly irrelevant studies, and elimination of 69 records with inappropriate publication types, 180 articles remained for title and abstract screening. Of these, 109 records were excluded, including reviews or systematic reviews (n = 38), case reports (n = 24), dissertations or theses (n = 22), and single arm studies (n = 25), leaving 71 articles for full text assessment (Figure 1). Full text evaluation excluded 56 studies because of incomplete outcome or baseline data (n = 18), non-comparative or single-group designs without a concurrent control arm (n = 16), mismatched interventions (n = 12), or failure to satisfy other predefined eligibility criteria (n = 10). Comparative observational cohort studies meeting prespecified eligibility criteria were retained. Ultimately, 15 comparative studies (Lu et al., 2022; Yao et al., 2025; Yang et al., 2025; Sun et al., 2025; Zhu, 2025; Feng et al., 2024; Zhang et al., 2024; Zeng and Wang, 2024; Wang and Wang, 2024; Du, 2024; Li et al., 2023; Meng et al., 2023; Yang et al., 2023; Dong, 2023; Han et al., 2022) published between 2022 and 2025 fulfilled the eligibility criteria and were included in the qualitative and quantitative syntheses (Figure 1).

### Characteristics of included studies

The baseline characteristics of the included studies are summarized in Tables 1 and 2. Fifteen comparative studies (Lu et al., 2022; Yao et al., 2025; Yang et al., 2025; Sun et al., 2025; Zhu, 2025; Feng et al., 2024; Zhang et al., 2024; Zeng and Wang, 2024; Wang and Wang, 2024; Du, 2024; Li et al., 2023; Meng et al., 2023; Yang et al., 2023; Dong, 2023; Han et al., 2022) published between 2022 and 2025 were included, comprising 1,902 patients, with individual sample sizes ranging from 40 (Du, 2024) to 429 (Lu et al., 2022) participants (Table 1). Fourteen studies were conducted at a single center, whereas one was a multicenter phase III randomized controlled trial involving 53 centers (Lu et al., 2022). The study populations consisted predominantly of patients with stage III or IV non small cell lung cancer harboring EGFR driver mutations. Reported mean or median age generally ranged from approximately 50–66 years, and both male and female patients were represented across all studies. Smoking history was available in most studies, although several reports did not provide detailed smoking information. Most studies enrolled patients with advanced disease, while two studies focused exclusively on stage III disease (Zeng and Wang, 2024; Dong, 2023) and one study included patients with stage III to IV disease without reporting stage specific distributions (Zhu, 2025) (Table 1). Baseline characteristics were generally comparable between intervention and control groups within individual studies (Table 2). Almonertinib or almonertinib containing regimens were evaluated against active comparator therapies, including osimertinib, furmonertinib, gefitinib, icotinib, afatinib based combination therapy, or chemoradiotherapy. Sample sizes in the intervention and control groups ranged from 20 to 214 and from 20 to 215 patients (Lu et al., 2022; Du, 2024), respectively. All included participants had EGFR mutation positive disease, including sensitizing mutations, exon 19 deletion, L858R mutation, T790M mutation, or other documented EGFR driver alterations. Age, sex distribution, and TNM stage were generally balanced between treatment groups within each study, indicating good baseline comparability for pooled analyses (Table 2).

**TABLE 1:** Pooled baseline characteristics of included studies.

Study	Year	Study center	Total participants	Sex (n)		Age	Smoking status (n)		TNM stage (n)			Remarks
				Male	Female		Smokers	Non-smokers	Stage II	Stage III	Stage IV	
(Yao et al., 2025)	2025	Single-center	200	76	124	62.28 ± 9.12	32	168	0	6	194	Original report: stage IIIB/IV; IIIB counted as stage III (n = 6)
(Yang et al., 2025)	2025	Single-center	104	43	61	<60 years: 45; ≥60 years: 59	30	74	0	28	76	Age reported by strata; mean ± SD not reported. Stage III corresponds to stage III in the original article
(Sun et al., 2025)	2025	Single-center	94	50	44	59.39 ± 8.84	-	-	0	16	78	Stage III corresponds to stage IIIB in the original article
(Zhu, 2025)	2025	Single-center	118	66	52	59.25 ± 7.94	-	-	-	-	-	Patients with stage III–IV were enrolled; stage-specific counts were not reported
(Feng et al., 2024)	2024	Single-center	137	55	82	63.55 ± 9.93	29	108	0	103	34	Stage III corresponds to stage IIIB in the original article
(Zhang et al., 2024)	2024	Single-center	64	26	38	63.11 ± 8.37	-	-	0	14	50	Stage III corresponds to stage IIIB in the original article
(Zeng and Wang, 2024)	2024	Single-center	100	49	51	50–70 (60.88 ± 3.49)	57	43	0	100	0	All enrolled patients had locally advanced stage III NSCLC.
(Wang and Wang, 2024)	2024	Single-center	78	43	35	50.61 ± 6.21	-	-	-	-	-	TNM stage distribution was not reported in the original article
(Du, 2024)	2024	Single-center	40	23	17	50–73 (61.80 ± 5.95)	-	-	-	-	-	TNM stage distribution was not reported in the original article
(Li et al., 2023)	2023	Single-center	160	52	108	50 (41–60)	50	110	18^*^	32	110	Age reported as median (range). Stage III corresponds to stage IIIB.
(Meng et al., 2023)	2023	Single-center	128	50	78	62.25 ± 8.32	47	81	-	-	-	TNM stage distribution was not reported in the baseline table
(Yang et al., 2023)	2023	Single-center	92	65	27	56.55 ± 7.47	69	23	0	30	62	Final analysis included 92 patients. Stage III corresponds to stage IIIB.
(Dong, 2023)	2023	Single-center	60	36	24	65.69 ± 4.94	-	-	0	60	0	All enrolled patients had inoperable stage III EGFR-mutant NSCLC.
(Lu et al., 2022)	2022	Multicenter (53 centers)	429	160	269	Median 59/62; range 25–81	129	300	0	29	400	AENEAS trial. Smokers = former +current smokers. Stage III corresponds to stage IIIB.
(Han et al., 2022)	2022	Single-center	98	68	30	58.01 ± 8.37	63	35	0	35	63	Stage III corresponds to clinical stage III in the original article

This table summarizes pooled baseline data for the overall study population in each article, without intervention-group or control-group details. Studies are listed by publication year from newest to oldest. “-” indicates data not reported in the original article; “*” indicates recurrence; specific TNM, staging is not available. Stage IIIB, was classified as stage III., Age is reported as mean ± SD, when available; otherwise, median, range, or stratified data from the original article are shown. TNM, the TNM, staging system, jointly developed and maintained by the Union for International Cancer Control (UICC) and the American Joint Committee on Cancer (AJCC), is the globally accepted standard for classifying the extent of malignant tumors. This anatomically-based system categories cancer progression into three benchmarking components: T (Tumor) representing the size and local extent of the primary tumor; N (Node) indicating the presence, number, or location of regional lymph node metastases; and M (Metastasis) denoting the presence or absence of distant metastasis.

**TABLE 2:** Summary of study design, grouping, and patient baseline characteristics.

Study	Year	Sample size (intervention/control)	Age, years (mean ± SD or range)		Sex (male/female, n)		TNM stage (II/III/IV, n)		EGFR driver mutation Positive
			Intervention	Control	Intervention	Control	Intervention	Control	
(Yao et al., 2025)	2025	79/121	62.82 ± 9.34	61.93 ± 8.99	32/47	44/77	0/5/74	0/1/120	L858R mutation (+)
(Yang et al., 2025)	2025	52/52	<60 y: 25; ≥60 y: 27	<60 y: 20; ≥60 y: 32	21/31	22/30	0/12/40	0/16/36	EGFR sensitizing/T790M mutation (+)
(Sun et al., 2025)	2025	47/47	58.64 ± 9.16	60.13 ± 8.54	26/21	24/23	0/7/40	0/9/38	EGFR driver mutation (+)
(Zhu, 2025)	2025	59/59	59.4 ± 8.6	59.1 ± 7.3	34/25	32/27	—	—	EGFR driver mutation (+)
(Feng et al., 2024)	2024	72/65	63.33 ± 10.15	63.80 ± 9.76	29/43	26/39	0/54/18	0/49/16	EGFR mutation (+)
(Zhang et al., 2024)	2024	32/32	62.34 ± 8.35	63.88 ± 8.45	14/18	12/20	0/6/26	0/8/24	EGFR exon 19/21 mutation (+)
(Zeng and Wang, 2024)	2024	57/43	61.23 ± 3.25 (50–70)	60.47 ± 3.78 (50–70)	29/28	20/23	0/57/0	0/43/0	EGFR exon 19 deletion (+)
(Wang and Wang, 2024)	2024	39/39	51.15 ± 6.24	50.08 ± 6.21	21/18	22/17	—	—	EGFR driver mutation (+)
(Du, 2024)	2024	20/20	62.01 ± 6.21	61.58 ± 5.83	12/8	11/9	—	—	EGFR sensitizing mutation (+)
(Li et al., 2023)	2023	80/80	51 (41–60)	50 (41–59)	28/52	24/56	8^*^/16/56	10^*^/16/54	EGFR T790M mutation (+)
(Meng et al., 2023)	2023	64/64	61.4 ± 8.6	63.1 ± 8.0	27/37	23/41	—	—	EGFR T790M mutation (+)
(Yang et al., 2023)	2023	46/46	57.39 ± 6.70	55.72 ± 8.15	31/15	34/12	0/16/30	0/14/32	EGFR mutation (+)
(Dong, 2023)	2023	30/30	65.28 ± 4.63	66.11 ± 5.28	17/13	19/11	0/30/0	0/30/0	EGFR mutation (+)
(Lu et al., 2022)	2022	214/215	32–78 (median 59)	25–81 (median 62)	80/134	80/135	0/12/202	0/17/198	EGFR sensitizing mutation (+)
(Han et al., 2022)	2022	49/49	59.88 ± 7.51	56.13 ± 8.84	32/17	36/13	0/19/30	0/16/33	EGFR sensitizing mutation (+)

The intervention group refers to almonertinib (or almonertinib-containing) regimens and the control group to the corresponding comparator. Sample size is shown as intervention/control. Age data are reported as originally published for each group, preferably as mean ± SD; otherwise as median (range) or stratified data. Sex is shown as male/female (n). TNM, stage is shown as stage II/III/IV (n); stage IIIB, was classified as stage III; “—” indicates not reported. All patients met inclusion criteria for EGFR, driver-gene-positive disease. “*” indicates recurrence; specific TNM, staging is not available.

Abbreviations: EGFR, epidermal growth factor receptor; EGFR-TKI, epidermal growth factor receptor tyrosine kinase inhibitor; TNM, tumor-node-metastasis.

### Treatment characteristics and outcome reporting

The treatment characteristics and reported outcome measures of the included studies are summarized in Table 3. Across all 15 comparative studies, almonertinib was administered orally at a uniform dose of 110 mg once daily, either as monotherapy or in combination with chemotherapy, bevacizumab, or radiotherapy, depending on the study design (Supplementary Figure 1; Table 3). Comparator regimens included osimertinib in six studies (Yao et al., 2025; Yang et al., 2025; Zhu, 2025; Feng et al., 2024; Li et al., 2023; Meng et al., 2023), gefitinib in three studies (Lu et al., 2022; Zeng and Wang, 2024; Wang and Wang, 2024), furmonertinib in two studies (Sun et al., 2025; Zhang et al., 2024), icotinib in two studies (Du, 2024; Han et al., 2022), afatinib combined with bevacizumab in one study (Yang et al., 2023), and chemoradiotherapy without EGFR tyrosine kinase inhibitor treatment in one study (Dong, 2023) (Supplementary Figure 1; Table 3). Treatment duration varied according to study protocol, including fixed treatment courses of two to six cycles, treatment for 3 months, or continuous administration until disease progression, relapse, death, withdrawal of consent, intolerable toxicity, or the end of follow up. All included studies reported at least two predefined efficacy or safety outcomes. Disease control rate and the overall adverse drug reaction profile were each reported in 14 studies (93.3%), objective response rate and rash incidence in 13 studies (86.7%), grade 3 adverse drug reactions in 10 studies (66.7%), diarrhea incidence in 8 studies (53.3%), and nausea or vomiting incidence in 7 studies (46.7%) (Supplementary Figure 1; Table 3). The broad and consistent reporting of efficacy and safety endpoints across studies provided sufficient outcome data for subsequent quantitative synthesis.

**TABLE 3:** Basic characteristics of comparative studies of almonertinib.

Study	Year	Intervention /control (n)	Sex (male/female)	KPS/ECOG/QOLetc.	Intervention		Treatment duration	Outcome endpoints
					Intervention	Control		
(Yao et al., 2025)	2025	79/121	32/47; 44/77	ECOG 0/1/2: 60/19/1 vs. 93/27/0	Almonertinib 110 mg po qd	Osimertinib 80 mg po qd	Until disease progression or last follow-up	cdefg
(Yang et al., 2025)	2025	52/52	21/31; 22/30	PS ≤ 2	Almonertinib 110 mg po qd	Osimertinib 80 mg po qd	Until disease progression or intolerable toxicity	abcdefg
(Sun et al., 2025)	2025	47/47	26/21; 24/23	ECOG 0–2	Almonertinib 110 mg po qd ± pemetrexed + cisplatin	Furmonertinib 80 mg po qd ± pemetrexed + cisplatin	2 cycles (21 d/cycle) plus continued TKI	abcdg
(Zhu, 2025)	2025	59/59	34/25; 32/27	—	Pemetrexed + cisplatin + almonertinib 110 mg po qd	Pemetrexed + cisplatin + osimertinib 80 mg po qd	4 cycles (21 d/cycle)	abdg
(Feng et al., 2024)	2024	72/65	29/43; 26/39	—	Almonertinib 110 mg po qd (second-line)	Osimertinib 80 mg po qd (first-line)	21 d/cycle for 3 cycles or until progression	abcdefg
(Zhang et al., 2024)	2024	32/32	14/18; 12/20	KPS ≥70	Almonertinib 110 mg po qd ± chemotherapy	Furmonertinib 80 mg po qd ± chemotherapy	Until disease progression	abcdefg
(Zeng and Wang, 2024)	2024	57/43	29/28; 20/23	—	Almonertinib 110 mg po qd	Gefitinib 250 mg po qd	2 cycles (28 d/cycle)	abfg
(Wang and Wang, 2024)	2024	39/39	21/18; 22/17	—	Almonertinib 110 mg po qd	Gefitinib 250 mg po qd	3 months	abcdeg
(Du, 2024)	2024	20/20	12/8; 11/9	—	Almonertinib 110 mg po qd	Icotinib 125 mg po tid	21 d/cycle for 3 cycles	abcdg
(Li et al., 2023)	2023	80/80	28/52; 24/56	ECOG 0/1: 32/48 vs. 30/50	Almonertinib 110 mg po qd	Osimertinig po qd	From first dose until relapse or death	abcdefg
(Meng et al., 2023)	2023	64/64	27/37; 23/41	—	Almonertinib 110 mg po qd (sequential)	Osimertinib 80 mg po qd (sequential)	Until progression, death, or end of follow-up	abfg
(Yang et al., 2023)	2023	46/46	31/15; 34/12	ECOG 0–1	Bevacizumab 400 mg iv d1 + almonertinib 110 mg po qd	Bevacizumab 400 mg iv d1 + afatinib 40 mg po qd	3 cycles (21 d/cycle)	abcdeg
(Dong, 2023)	2023	30/30	17/13; 19/11	—	PP chemotherapy + radiotherapy + almonertinib 110 mg po qd	PP chemotherapy + radiotherapy (no TKI)	6 chemotherapy cycles (21 d/cycle) plus TKI until progression	abefg
(Lu et al., 2022)	2022	214/215	80/134; 80/135	ECOG 0/1: 51/160 vs. 54/159	Aumolertinib 110 mg po qd	Gefitinib 250 mg po qd	Until progression, consent withdrawal, or intolerable toxicity	abcdfg
(Han et al., 2022)	2022	49/49	32/17; 36/13	ECOG 0–1	Almonertinib 110 mg po qd	Icotinib 125 mg po tid	2 cycles (21 d/cycle; ∼63 d total)	abcdeg

Studies are listed by publication year (newest to oldest). The Study column shows the full name of the first author only. Sample size is shown as total/intervention/control. Age and sex are reported as originally published for each group; “—” indicates not reported. Outcome endpoints indicate whether each study reported the corresponding outcome (not the numeric results): a, objective response rate (ORR); b, disease control rate (DCR); c, incidence of diarrhea; d, incidence of rash; e, incidence of nausea and vomiting; f, number of patients with grade ≥3 adverse drug reactions (ADRs); g, overall ADR, profile. “—” indicates no relevant data.

Abbreviations: ADR, adverse drug reaction; AE, adverse event; DCR, disease control rate; ECOG, eastern cooperative oncology group; EGFR, epidermal growth factor receptor; EGFR-TKI, epidermal growth factor receptor tyrosine kinase inhibitor; KPS, karnofsky performance status; ORR, objective response rate; PP, pemetrexed plus cisplatin; PS, performance status; QOL, quality of life; RECIST, response evaluation criteria in solid tumors; TKI, tyrosine kinase inhibitor; TNM, tumor-node-metastasis.

### Methodological quality and certainty of evidence

Among the 15 comparative studies, methodological quality was generally acceptable, with most investigations employing predefined comparative designs and standard statistical approaches for efficacy and survival evaluation (Figures 2A–D; Table 4). The studies published in 2025 comprised two retrospective comparative cohorts (Yao et al., 2025; Yang et al., 2025), and two open-label randomized controlled trials (Sun et al., 2025; Zhu, 2025), Studies published in 2024 included one prospective nonrandomized cohort (Feng et al., 2024), three open-label randomized controlled trials (Zhang et al., 2024; Wang and Wang, 2024; Du, 2024), and one observational cohort (Zeng and Wang, 2024). Most studies incorporated established statistical methods, including Kaplan–Meier analysis, Cox regression, log rank testing, and chi square comparisons, supporting the reliability of outcome assessment (Table 4). Among studies published in 2023, three randomized trials (Meng et al., 2023; Yang et al., 2023; Dong, 2023) and one retrospective cohort study (Li et al., 2023) were included. The 2022 publications consisted of the multicenter AENEAS phase III trial (Lu et al., 2022) and one open-label randomized trial (Han et al., 2022). Notably, the AENEAS phase III trial (Lu et al., 2022) represented the methodological benchmark of the evidence base, being the only multicenter double blind randomized study and the only trial incorporating centralized randomization, intention to treat analysis, and formal sample size estimation. Another study also adopted a randomized design, further contributing prospective comparative evidence to the pooled dataset (Han et al., 2022) (Figures 2B–D). Overall, random sequence generation was reported in one-third of studies (33.3%), while outcome data were adequately addressed in 80.0% of investigations (Figure 2A). Although allocation concealment and blinding were incompletely reported in several single center studies, nearly half of the included studies achieved moderate certainty ratings (46.7%), and one large phase III multicenter trial provided high certainty evidence. Collectively, these findings indicate that the current evidence base is supported by a substantial proportion of randomized studies, complemented by real world observational data that may enhance the generalizability of clinical findings (Figures 2C,D).

**FIGURE 2:**
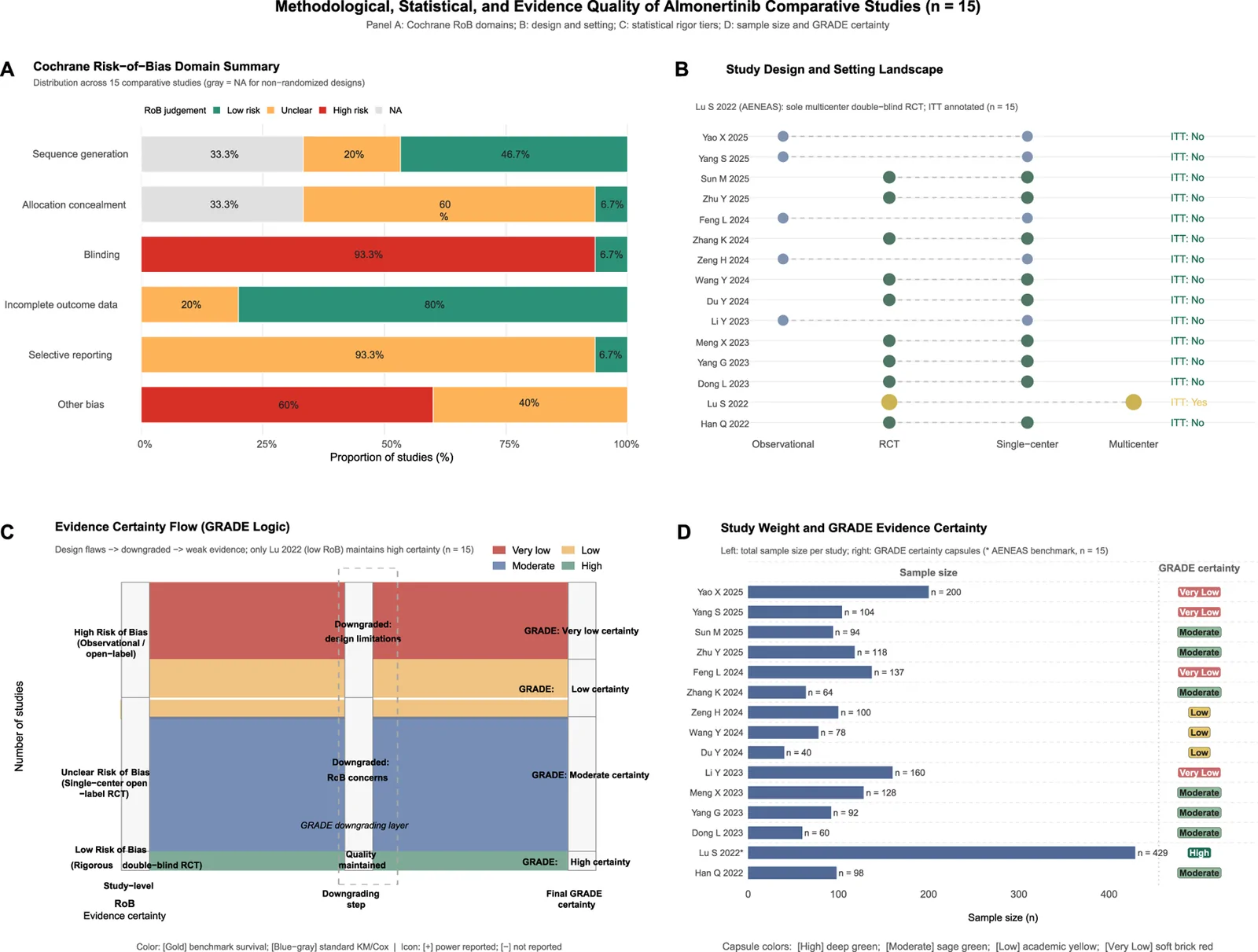
Methodological, statistical, and evidence quality of the 15 comparative studies evaluating almonertinib **(A)** Cochrane risk of bias domain summary showing the proportional distribution of low, unclear, high, and not applicable judgments across sequence generation, allocation concealment, blinding, incomplete outcome data, selective reporting, and other bias domains **(B)** study design and setting landscape showing whether each study was observational or randomized, single center or multicenter, and whether intention to treat analysis was reported **(C)** GRADE evidence certainty flow illustrating how study level risk of bias, design limitations, and downgrading decisions contributed to final certainty ratings from very low to high **(D)** study level sample size distribution and corresponding GRADE certainty classification, showing the relative scale and evidence certainty of each included study, with the AENEAS trial highlighted as the multicenter double blind randomized benchmark. Abbreviations: AENEAS, a phase III multicenter double blind randomized controlled trial evaluating almonertinib in patients with EGFR mutated non small cell lung cancer; Cox, Cox proportional hazards regression; EGFR, epidermal growth factor receptor; GRADE, Grading of Recommendations Assessment, Development and Evaluation; ITT, intention to treat; KM, Kaplan Meier; n, number of participants or number of studies; NA, not applicable; NSCLC, non small cell lung cancer; RECIST, Response Evaluation Criteria in Solid Tumors; RCT, randomized controlled trial; RoB, risk of bias.

**TABLE 4:** Methodological and statistical quality of comparative studies.

Study	Year	Study design	Setting	Methodological quality (Cochrane RoB domains)						ITT analysis	Statistical design		Overall RoB	GRADE certainty
				Sequence generation	Allocation concealment	Blinding	Incomplete outcome	Selective reporting	Other bias		Sample size estimation	Main statistical methods		
(Yao et al., 2025)	2025	Retrospective comparative cohort	Single-center	NA	NA	No	Unclear	Unclear	Selection bias (non-random treatment assignment)	No	No	Kaplan-Meier, log-rank, Cox regression; χ^2^/Fisher	High	Very low
(Yang et al., 2025)	2025	Retrospective comparative cohort	Single-center	NA	NA	No	Unclear	Unclear	Selection and confounding bias	No	No	Cox regression; χ^2^; survival analysis	High	Very low
(Sun et al., 2025)	2025	Open-label RCT	Single-center	Random number table	Unclear	No	Yes	Unclear	Optional chemotherapy co-intervention	No	No	SPSS 28.0; t-test, χ^2^, Fisher; Log-rank	Unclear	Moderate
(Zhu, 2025)	2025	Open-label RCT	Single-center	Random number table	Unclear	No	Yes	Unclear	Unclear	No	No	χ^2^; t-test	Unclear	Moderate
(Feng et al., 2024)	2024	Prospective non-randomized cohort	Single-center	NA	NA	No	Yes	Unclear	Confounding (non-parallel 2L vs. 1L comparison)	No	No	SPSS 28.0; t-test, χ^2^	High	Very low
(Zhang et al., 2024)	2024	Open-label RCT	Single-center	Random number table	Unclear	No	Yes	Unclear	Unclear	No	No	χ^2^; survival analysis	Unclear	Moderate
(Zeng and Wang, 2024)	2024	Observational cohort	Single-center	NA	NA	No	Unclear	Unclear	Selection bias (grouped by treatment choice)	No	No	χ^2^; survival curves	High	Low
(Wang and Wang, 2024)	2024	Open-label RCT	Single-center	Computer-generated randomization	Unclear	No	Yes	Unclear	Ethics approval not clearly reported	No	No	SPSS 20.0; t-test, χ^2^	Unclear	Low
(Du, 2024)	2024	Open-label RCT (retrospective enrollment)	Single-center	Random (method unclear)	Unclear	No	Yes	Unclear	Retrospective patient selection	No	No	χ^2^; t-test	High	Low
(Li et al., 2023)	2023	Retrospective comparative cohort	Single-center	NA	NA	No	Yes	Unclear	Treatment assignment by clinical practice	No	No	Kaplan-Meier, log-rank; Mann-Whitney, χ^2^; SPSS 21.0/Prism 8	High	Very low
(Meng et al., 2023)	2023	Open-label RCT	Single-center	Stratified random (software-generated)	Unclear	No	Yes	Unclear	Unclear	No	No	SPSS 26.0; χ^2^, rank-sum; Kaplan-Meier	Unclear	Moderate
(Yang et al., 2023)	2023	Open-label RCT	Single-center	Random (method unclear)	Unclear	No	Yes	Unclear	5 early dropouts (per-protocol analysis)	No	No	t-test, χ^2^	Unclear	Moderate
(Dong, 2023)	2023	Open-label RCT	Single-center	Random number table	Unclear	No	Yes	Unclear	Small sample (n = 60)	No	No	SPSS 23.0; t-test, χ^2^	Unclear	Moderate
(Lu et al., 2022)	2022	Phase III double-blind RCT (AENEAS)	Multicenter (53 sites)	IWRS central randomization	Adequate	Yes	Yes	No	Industry-sponsored trial	Yes	Yes (90% power; NCT03849768)	SAS 9.4; stratified Cox, Kaplan-Meier, logistic regression	Low	High
(Han et al., 2022)	2022	Open-label RCT	Single-center	Random assignment (method unclear)	Unclear	No	Yes	Unclear	Unclear	No	No	RECIST 1.1; χ^2^; PFS analysis	Unclear	Moderate

Studies are listed by publication year (newest to oldest). Methodological quality was assessed using Cochrane risk-of-bias (RoB) domains for randomized trials and analogous principles for observational studies (non-randomized domains marked NA). “Yes” under Incomplete outcome data indicates low risk of attrition bias (complete or adequately handled outcome data); “No” indicates high risk. Overall RoB reflects the integrated judgment across domains. ITT, intention-to-treat. GRADE, certainty reflects the overall confidence that the study evidence supports clinical recommendations, rated High, Moderate, Low, or Very low per the GRADE, Working Group; RCTs, started at High and were downgraded for RoB, imprecision, indirectness, or other limitations; observational studies started at Low/Very low. Only Lu 2022 (AENEAS, phase III, double-blind multicenter RCT) achieved High certainty; most single-center open-label RCTs, were Moderate; retrospective or non-randomized studies were Low or Very low. Abbreviations used in the table are defined below. Statistical software and tests were extracted from original Methods sections.

Abbreviations: CI, confidence interval; Cox, Cox proportional hazards regression; DCR, disease control rate; ECOG, eastern cooperative oncology group; EGFR, epidermal growth factor receptor; FAS, full analysis set; Fisher, Fisher exact test; GRADE, grading of recommendations assessment, Development and Evaluation; ITT, intention-to-treat; IWRS, interactive web response system; Kaplan-Meier, Kaplan-Meier survival analysis; Log-rank, log-rank test; NA, not applicable; NGS, next-generation sequencing; NSCLC, non-small cell lung cancer; ORR, objective response rate; PFS, progression-free survival; RCT, randomized controlled trial; RECIST, response evaluation criteria in solid tumors; RoB, risk of bias; SAS, SAS statistical software; SPSS, statistical package for the social sciences; TKI, tyrosine kinase inhibitor; χ^2^, chi-square test.

### Meta analysis of objective response rate and disease control rate

Objective response rate and disease control rate data extracted from the included comparative studies are summarized in Table 5. Fourteen studies reported both ORR and DCR and were eligible for quantitative synthesis, whereas one study was excluded because neither response outcome was available (Yao et al., 2025) (Table 5). For ORR, a random effects model was applied because moderate heterogeneity was observed among studies (I^2^ = 65.7%, P = 0.0003). The pooled analysis demonstrated that almonertinib based treatment significantly improved objective response rate compared with the control regimens (RR = 1.33, 95% CI 1.08 to 1.65, P = 0.0077) (Figure 3A). Significant improvements were observed in five studies (Du, 2024; Li et al., 2023; Yang et al., 2023; Dong, 2023; Han et al., 2022), whereas the remaining studies showed no statistically significant difference between treatment groups. No study demonstrated a significant reduction in objective response rate with almonertinib treatment. For DCR, substantial between study heterogeneity was identified (I^2^ = 74.7%, P < 0.0001), and a random effects model was therefore adopted. The pooled estimate demonstrated a significant improvement in disease control rate with almonertinib based therapy compared with the control group (RR = 1.14, 95% CI 1.03 to 1.26, P = 0.0099) (Figure 3B). Significant advantages were observed in seven studies (Zeng and Wang, 2024; Wang and Wang, 2024; Du, 2024; Li et al., 2023; Yang et al., 2023; Dong, 2023; Han et al., 2022), whereas the remaining studies showed comparable disease control between treatment groups. Overall, the pooled analyses consistently favored almonertinib for both objective response rate and disease control rate despite moderate to substantial heterogeneity across studies.

**TABLE 5:** Efficacy outcomes of comparative studies of almonertinib.

Study	Year	Tumor response per RECIST 1.1								ORR		DCR		mPFS (months)		mOS (months)	
		CR		PR		SD		PD									
		Intervention	Control	Intervention	Control	Intervention	Control	Intervention	Control	Intervention	Control	Intervention	Control	Intervention	Control	Intervention	Control
(Yao et al., 2025)	2025	—	—	—	—	—	—	—	—	—	—	—	—	19.4	18.5	—	—
(Yang et al., 2025)	2025	1 (1.92%)	1 (1.92%)	10 (19.23%)	5 (9.62%)	37 (71.15%)	41 (78.85%)	4 (7.69%)	5 (9.62%)	11 (21.15%)	6 (11.54%)	48 (92.31%)	47 (90.38%)	16.0	12.0	27.0	18.0
(Sun et al., 2025)	2025	0	0	23 (48.94%)	26 (55.32%)	15 (31.91%)	14 (29.79%)	9 (19.15%)	7 (14.89%)	23 (48.94%)	26 (55.32%)	38 (80.85%)	40 (85.11%)	8.6	11.3	—	—
(Zhu, 2025)	2025	7 (11.9%)	8 (13.5%)	24 (40.7%)	25 (42.4%)	15 (25.4%)	14 (23.7%)	13 (22.0%)	12 (20.3%)	31 (52.5%)	33 (55.9%)	46 (78.0%)	47 (79.7%)	—	—	—	—
(Feng et al., 2024)	2024	0	0	31 (43.06%)	27 (41.54%)	35 (48.61%)	32 (49.23%)	6 (8.33%)	6 (9.23%)	31 (43.06%)	27 (41.54%)	66 (91.67%)	59 (90.77%)	—	—	—	—
(Zhang et al., 2024)	2024	0	0	19 (59.38%)	22 (68.75%)	1 (3.13%)	2 (6.25%)	12 (37.50%)	8 (25.00%)	19 (59.38%)	22 (68.75%)	20 (62.50%)	24 (75.00%)	4.0	11.9	12.2	20.5
(Zeng and Wang, 2024)	2024	0	0	16 (28.07%)	5 (11.63%)	28 (49.12%)	14 (32.56%)	13 (22.81%)	24 (55.81%)	16 (28.07%)	5 (11.63%)	44 (77.19%)	19 (44.19%)	5.8	4.0	16.9	10.5
(Wang and Wang, 2024)	2024	—	—	22 (56.41%)	20 (51.28%)	15 (38.46%)	10 (25.64%)	2 (5.13%)	9 (23.08%)	22 (56.41%)	20 (51.28%)	37 (94.87%)	30 (76.92%)	—	—	—	—
(Du, 2024)	2024	8 (40.00%)	3 (15.00%)	7 (35.00%)	5 (25.00%)	4 (20.00%)	6 (30.00%)	1 (5.00%)	6 (30.00%)	15 (75.00%)	8 (40.00%)	19 (95.00%)	14 (70.00%)	—	—	—	—
(Li et al., 2023)	2023	16 (20.0%)	12 (15.0%)	40 (50.0%)	26 (32.5%)	16 (20.0%)	24 (30.0%)	8 (10.0%)	18 (22.5%)	56 (70.0%)	38 (47.5%)	72 (90.0%)	62 (77.5%)	14.0	10.5	17.5	14.5
(Meng et al., 2023)	2023	—	—	—	—	—	—	—	—	42 (65.62%)	34 (53.12%)	53 (82.81%)	48 (75.00%)	11.76	11.02	—	—
(Yang et al., 2023)	2023	0	0	22 (47.83%)	6 (13.04%)	18 (39.13%)	23 (50.00%)	6 (13.04%)	17 (36.96%)	22 (47.83%)	6 (13.04%)	40 (86.96%)	29 (63.04%)	—	—	—	—
(Dong, 2023)	2023	5 (16.67%)	1 (3.33%)	11 (36.67%)	6 (20.00%)	12 (40.00%)	8 (26.67%)	2 (6.67%)	15 (50.00%)	16 (53.33%)	7 (23.33%)	28 (93.33%)	15 (50.00%)	11.26	9.25	21.23	16.52
(Lu et al., 2022)	2022	1 (0.5%)	1 (0.5%)	157 (73.4%)	154 (71.6%)	41 (19.2%)	53 (24.7%)	12 (5.6%)	6 (2.8%)	158 (73.8%)	155 (72.1%)	199 (93.0%)	208 (96.7%)	19.3	9.9	—	—
(Han et al., 2022)	2022	0	0	23 (46.93%)	7 (14.28%)	17 (34.69%)	20 (40.81%)	9 (18.36%)	22 (44.89%)	23 (46.93%)	7 (14.28%)	40 (81.63%)	27 (55.10%)	5.9	4.0	—	—

Intervention group = almonertinib or almonertinib-containing regimen; control group = comparator regimen. Studies are listed by publication year (newest to oldest). Sample size is intervention group/control group (Intervention/Control). The intervention group received almonertinib or an almonertinib-containing regimen; the control group received the comparator regimen stated in each source publication. Tumor response categories follow RECIST, 1.1: CR, complete response; PR, partial response (≥30% decrease in target lesions); SD, stable disease; PD, progressive disease (≥20% increase or new lesions). ORR = (CR + PR)/N×100%; DCR = (CR + PR + SD)/N×100%. mPFS/mOS, median progression-free/overall survival in months unless otherwise stated. “—” indicates not reported or not applicable. Calculated or derived values: Wang 2024 did not report CR, separately; ORR, and DCR, were calculated from PR, SD, and PD, counts (ORR = PR/N; DCR = [PR + SD]/N). Zhang 2024 DCR, was calculated from PR, and SD, counts. Li 2023 PD, counts were derived from N minus (CR + PR + SD). Han 2022 reported median PFS, as 179 days (intervention) and 121 days (control), converted to 5.9 and 4.0 months (÷30.4375). Yao 2025 and Meng 2023 did not report CR/PR/SD/PD, breakdown; Yao 2025 did not report ORR/DCR (primary endpoint was PFS). Lu 2022 (AENEAS) response data are from investigator-assessed best overall response; mOS, was immature at primary data cutoff and is not shown. Lu 2022 (AENEAS): CR + PR + SD + PD, did not sum to N in both arms because non-evaluable patients were excluded from the best overall response analysis (3/214 intervention; 1/215 control; verify against source publication). Sun M (Sun et al., 2025) reported 1-year survival rates (65.95% vs. 87.23% for almonertinib vs. furmonertinib) but did not report median OS., Feng 2024 is a non-parallel comparison (almonertinib second-line vs. osimertinib first-line). Dong 2023 control arm received chemoradiotherapy without TKI., Yang Guangxia 2023 intervention = bevacizumab plus almonertinib; control = bevacizumab plus afatinib.

Abbreviations: CR, complete response; DCR, disease control rate; FACT-L, Functional Assessment of Cancer Therapy–Lung; mOS, median overall survival; mPFS, median progression-free survival; N, number of patients; ORR, objective response rate; OS, overall survival; PD, progressive disease; PFS, progression-free survival; PR, partial response; QoL, quality of life; RECIST, response evaluation criteria in solid tumors; SAS, Self-Rating Anxiety Scale; SD, stable disease; SF-36, 36-Item Short Form Health Survey; SDS, Self-Rating Depression Scale.

**FIGURE 3:**
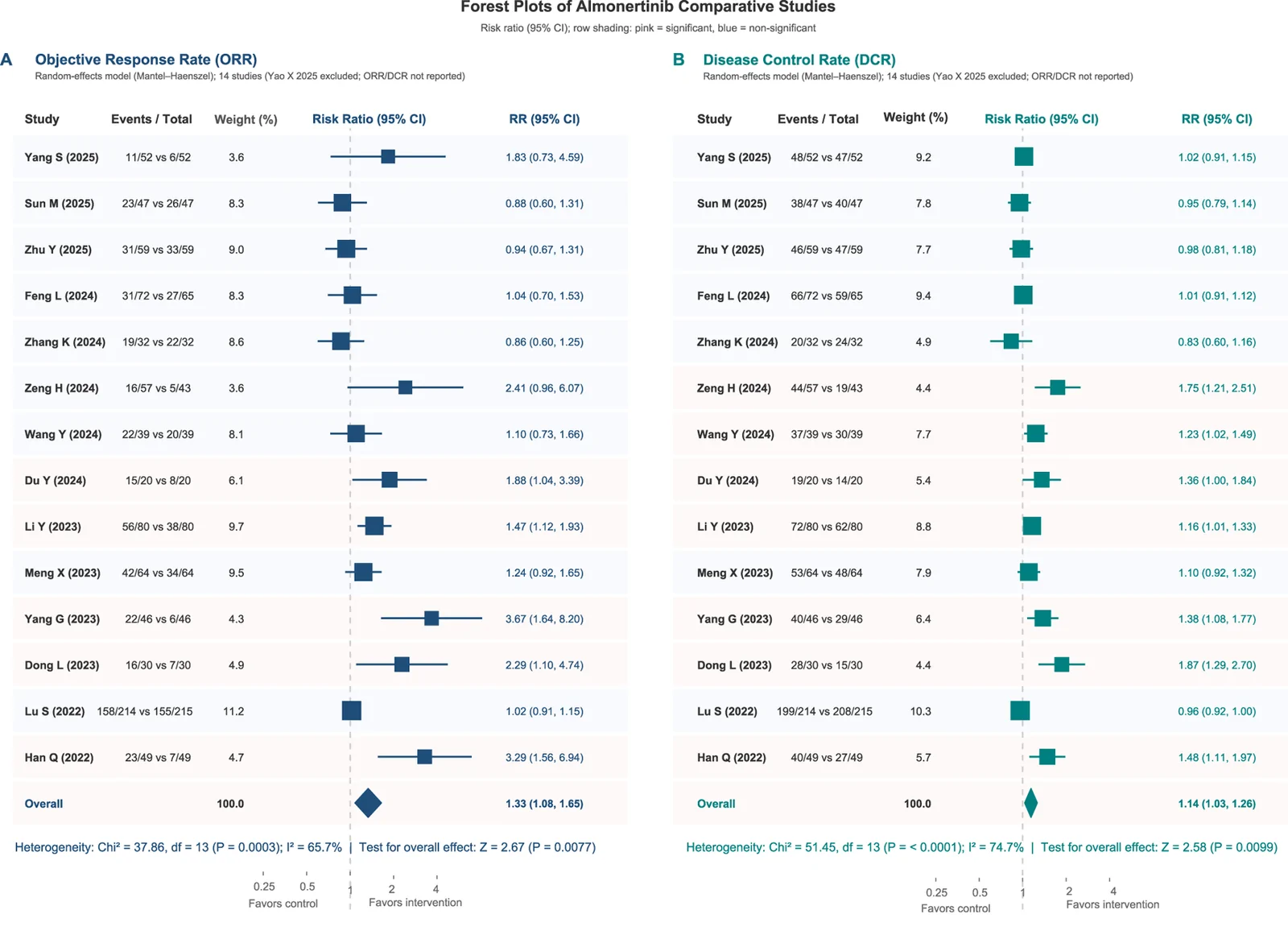
Forest plots of tumor response outcomes in comparative studies of almonertinib **(A)** Objective response rate was synthesized from 14 studies using a random effects Mantel Haenszel model, with Yao X 2025 excluded because ORR and DCR were not reported. Almonertinib based treatment was associated with a significantly higher ORR than control regimens, with a pooled RR of 1.33 (95% CI 1.08 to 1.65; P = 0.0077), although moderate heterogeneity was observed across studies (I^2^ = 65.7%) **(B)** Disease control rate was synthesized from the same 14 studies using a random effects Mantel Haenszel model. Almonertinib based treatment also showed a significant improvement in DCR compared with control regimens, with a pooled RR of 1.14 (95% CI 1.03 to 1.26; P = 0.0099), with substantial between study heterogeneity (I^2^ = 74.7%). In both panels, squares represent study specific effect estimates, horizontal lines indicate 95% CIs, diamond markers indicate pooled effects, and values greater than 1 favor almonertinib based treatment. Abbreviations: CI, confidence interval; DCR, disease control rate; df, degrees of freedom; I^2^, inconsistency statistic representing the percentage of total variation across studies attributable to heterogeneity rather than chance; Mantel Haenszel, a stratified method commonly used to pool dichotomous outcomes in meta analysis; ORR, objective response rate, defined as the proportion of patients achieving complete response or partial response according to tumor response criteria; P, probability value; RCT, randomized controlled trial; RECIST, Response Evaluation Criteria in Solid Tumors; RR, risk ratio.

Survival outcomes are summarized in Table 5 and Figure 4. Among the included studies, median progression free survival was reported in 10 studies, while median overall survival was available in 5 studies, with substantial variation in follow up duration across studies (Table 5). For median progression free survival, a random effects model was applied due to significant heterogeneity across studies (I^2^ = 83.3%, P < 0.001). The pooled analysis showed no statistically significant difference between almonertinib based therapy and control regimens (HR = 0.88, 95% CI 0.65 to 1.18, P = 0.395) (Figure 4A). At the individual study level, statistically significant prolongation of progression-free survival was observed in one study (Lu et al., 2022), whereas Yang S (Yang et al., 2025) and Zeng H (Zeng and Wang, 2024) showed numerical but non-significant PFS trends favoring almonertinib (95% CIs crossed 1.0). One study (Zhang et al., 2024) showed a significantly shorter progression-free survival with almonertinib compared with the control regimen. The remaining studies demonstrated no statistically significant differences between groups (Figure 4A). For median overall survival, a random effects model was also used owing to moderate heterogeneity (I^2^ = 64.6%, P = 0.023). The pooled estimate indicated no significant difference in overall survival between treatment groups (HR = 0.83, 95% CI 0.61 to 1.13, P = 0.247) (Figure 4B). At the study level, significant improvement in overall survival was observed in two studies (Yang et al., 2025; Zeng and Wang, 2024), while one study (Zhang et al., 2024) showed a statistically significant disadvantage for almonertinib. Other studies showed no significant differences in overall survival outcomes (Figure 4B). Overall, although some individual studies demonstrated benefit or disadvantage in survival endpoints, the pooled results did not show statistically significant differences in either progression free survival or overall survival between almonertinib based therapy and comparator regimens (Figures 4A,B).

**FIGURE 4:**
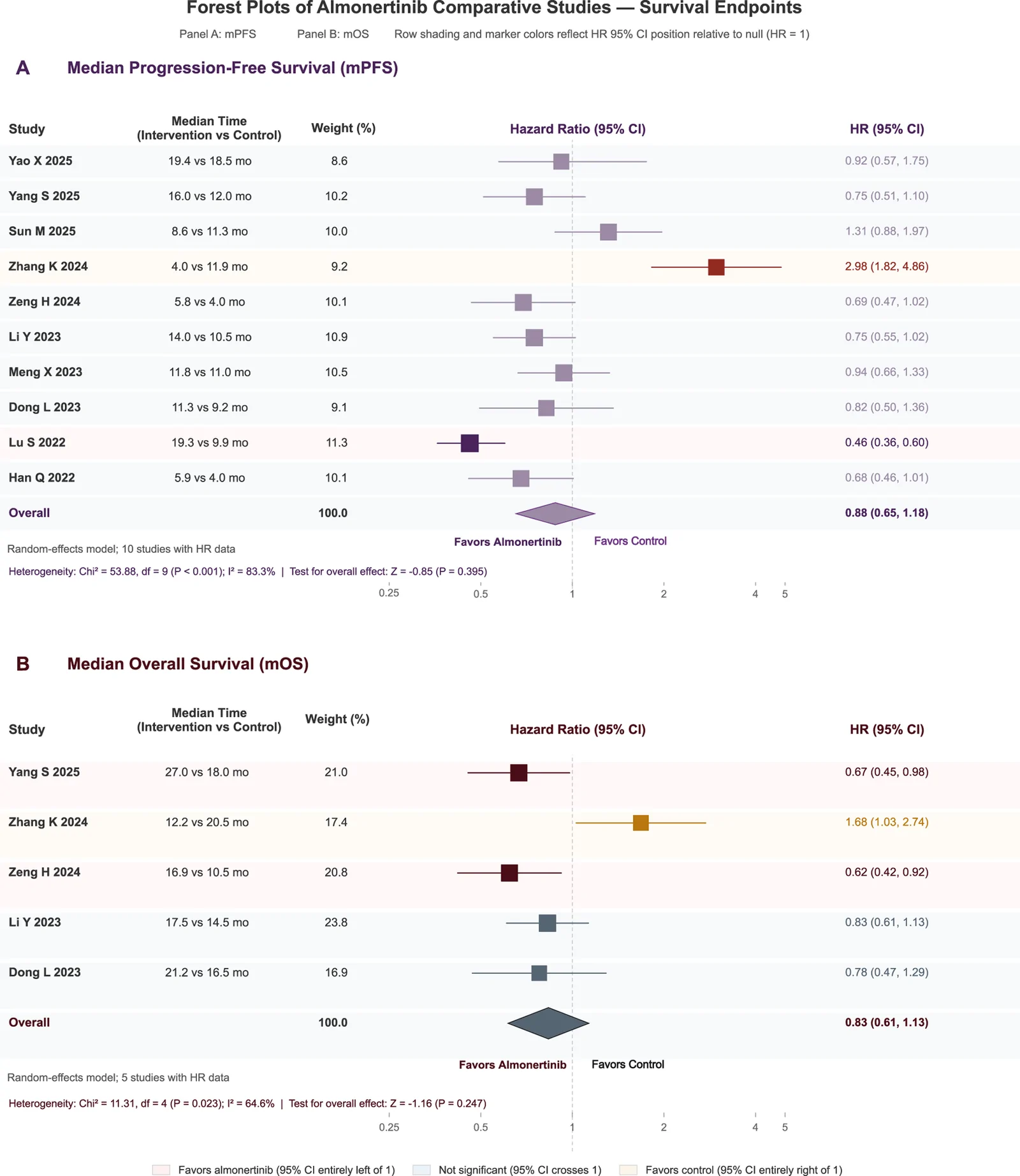
Forest plots of survival outcomes in comparative studies of almonertinib **(A)** Median progression free survival was analyzed across 10 studies using a random effects model, showing individual hazard ratios with corresponding 95% confidence intervals and pooled effect estimates, indicating no statistically significant difference between almonertinib based therapy and control regimens with substantial between study heterogeneity **(B)** Median overall survival was synthesized from 5 studies using a random effects model, also demonstrating no statistically significant difference between treatment groups, with moderate heterogeneity observed across studies. In both panels, effect sizes are presented as hazard ratios with values less than 1 favoring almonertinib and values greater than 1 favoring control, with square markers representing individual study weights and diamond markers indicating pooled estimates. Abbreviations: CI, confidence interval; df, degrees of freedom; HR, hazard ratio; I2, inconsistency index quantifying the proportion of total variability due to between study heterogeneity; mOS, median overall survival; mPFS, median progression free survival; P, probability value; RCT, randomized controlled trial; RECIST, Response Evaluation Criteria in Solid Tumors; SD, standard deviation; SE, standard error; TKI, tyrosine kinase inhibitor.

### Integrated ORR–DCR concordance and absolute treatment benefit

The integrated analysis of ORR and DCR across 14 comparative studies is summarized in Figure 5 and Supplementary Table 1. Most studies showed concordant relative effects between the two endpoints, while a small number showed concordance in the opposite direction (Sun et al., 2025; Zhu, 2025; Zhang et al., 2024), and only one study demonstrated discordance (Lu et al., 2022) (Supplementary Table 1). Overall pooled estimates indicated a consistent relative benefit favoring almonertinib across both endpoints. The dual endpoint analysis showed that most studies clustered in the upper right quadrant, indicating simultaneous improvement in ORR and DCR (Figure 5A). A positive association was observed between ln(RR) of ORR and DCR, with a regression slope of 0.393, suggesting partial concordance between the two treatment effects. The pooled estimate also supported a treatment favorable pattern (Figure 5A). Absolute effect analysis showed inter study variability in treatment benefit (Figure 5B). Several studies demonstrated greater ORR gain (Du, 2024; Yang et al., 2023; Dong, 2023; Han et al., 2022), while others showed more pronounced DCR improvement (Zeng and Wang, 2024; Wang and Wang, 2024; Dong, 2023). Most studies presented positive risk differences for both endpoints (Yang et al., 2025; Feng et al., 2024; Zeng and Wang, 2024; Wang and Wang, 2024; Du, 2024; Li et al., 2023; Meng et al., 2023; Yang et al., 2023; Dong, 2023; Han et al., 2022), whereas only a few showed minimal or negative effects (Lu et al., 2022; Sun et al., 2025; Zhu, 2025; Zhang et al., 2024). In some studies, DCR benefit exceeded ORR (Zhu, 2025; Zeng and Wang, 2024; Wang and Wang, 2024; Dong, 2023) suggesting broader disease stabilization beyond tumor shrinkage (Figure 5B). Overall, ORR and DCR showed consistent directional concordance with a moderate positive correlation and predominantly favorable absolute treatment effects, despite heterogeneity across studies (Supplementary Table 1; Figure 5).

**FIGURE 5:**
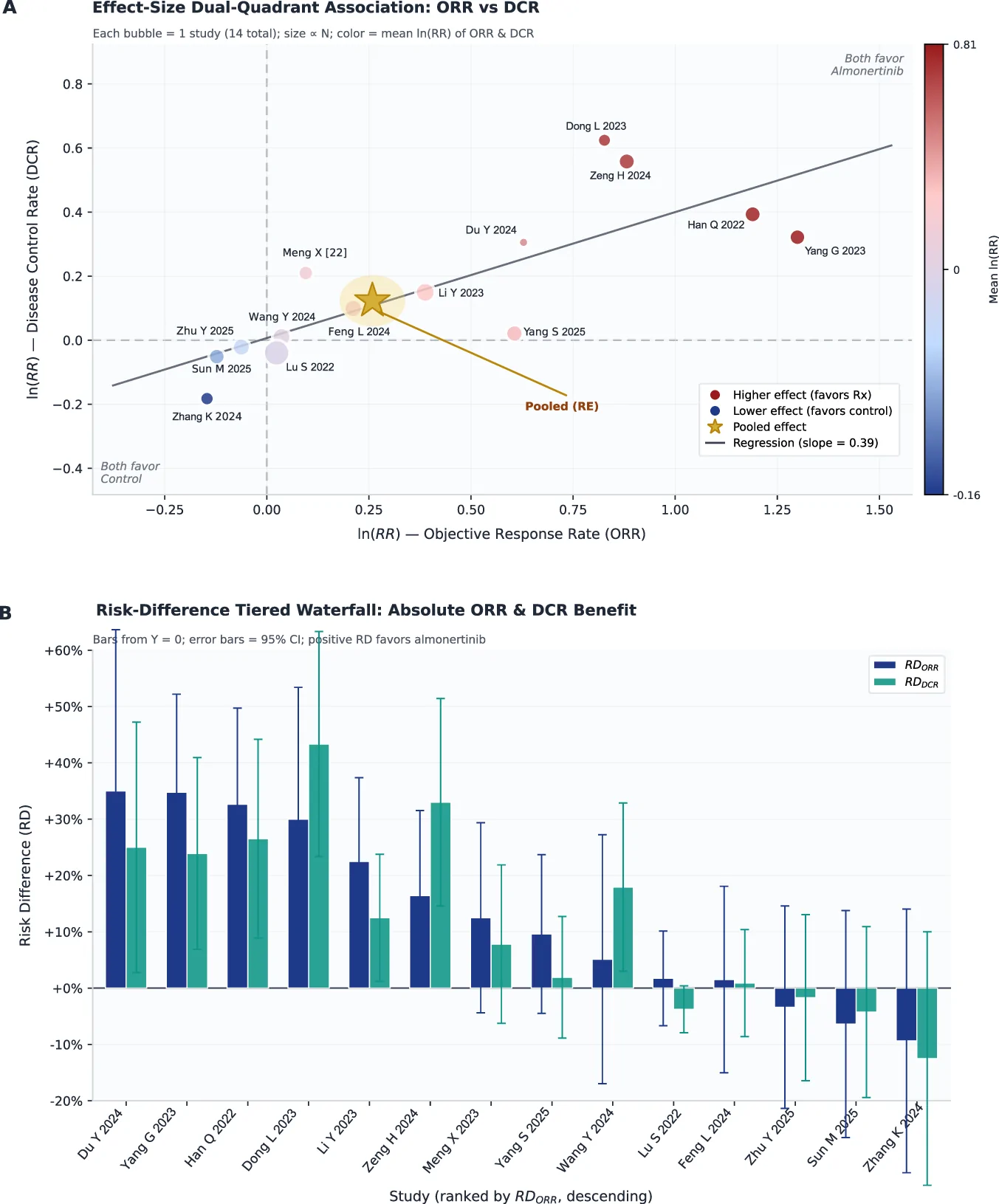
Integrated association between objective response rate and disease control rate effect sizes and absolute treatment benefit across comparative studies of almonertinib **(A)** Dual quadrant effect size association plot illustrating the relationship between log transformed risk ratios (ln(RR)) for objective response rate and disease control rate across 14 comparative studies, with each bubble representing one study, bubble size proportional to sample size and color reflecting mean ln(RR). The fitted regression line describes the cross endpoint relationship, while quadrant partitioning distinguishes concordant and discordant treatment effects, and the pooled estimate indicates the overall directional consistency of relative efficacy across endpoints **(B)** risk difference tiered waterfall plot presenting absolute treatment effects for objective response rate and disease control rate across studies ranked by RDORR in descending order, with bars representing risk differences from zero and whiskers indicating 95% confidence intervals, demonstrating inter study heterogeneity in absolute benefit and differences in magnitude between tumor response and disease control outcomes. Abbreviations: CI, confidence interval; DCR, disease control rate; ln(RR), natural logarithm of risk ratio; N, sample size; ORR, objective response rate; RD, risk difference; RE, random effects model; RR, risk ratio; Rx, almonertinib treatment arm.

### Subgroup analysis according to comparator regimens

Given the clinical heterogeneity of control regimens, comparator-stratified analyses were performed and are presented in Table 5 and Figure 6. Comparator treatments included osimertinib, gefitinib, furmonertinib, icotinib, and other regimens comprising afatinib combined with bevacizumab or chemoradiotherapy (Table 5). Particular attention was given to the comparison with osimertinib, which is most relevant to contemporary third-generation EGFR-TKI treatment selection. For ORR, the pooled estimate versus osimertinib was RR = 1.20 (95% CI 0.99–1.46) and did not reach statistical significance, although the point estimate favored almonertinib (Figure 6A). Statistically significant pooled ORR effects were observed in the icotinib subgroup (RR = 2.37, 95% CI 1.38–4.07) and the other comparator subgroup (RR = 2.83, 95% CI 1.65–4.86), whereas the gefitinib and furmonertinib subgroups were not statistically significant (Figure 6A). Heterogeneity within most comparator subgroups was low to moderate. A similar pattern was observed for DCR: significant pooled effects were identified in the icotinib subgroup (RR = 1.42, 95% CI 1.15–1.75) and the other comparator subgroup (RR = 1.55, 95% CI 1.16–2.07), whereas the remaining subgroup estimates, including that versus osimertinib, were not statistically significant (Figure 6B). These findings indicate that the overall response advantage should be interpreted in a comparator-specific manner rather than as a uniform effect against all control regimens. Formal statistical comparisons between comparator subgroups were not performed.

**FIGURE 6:**
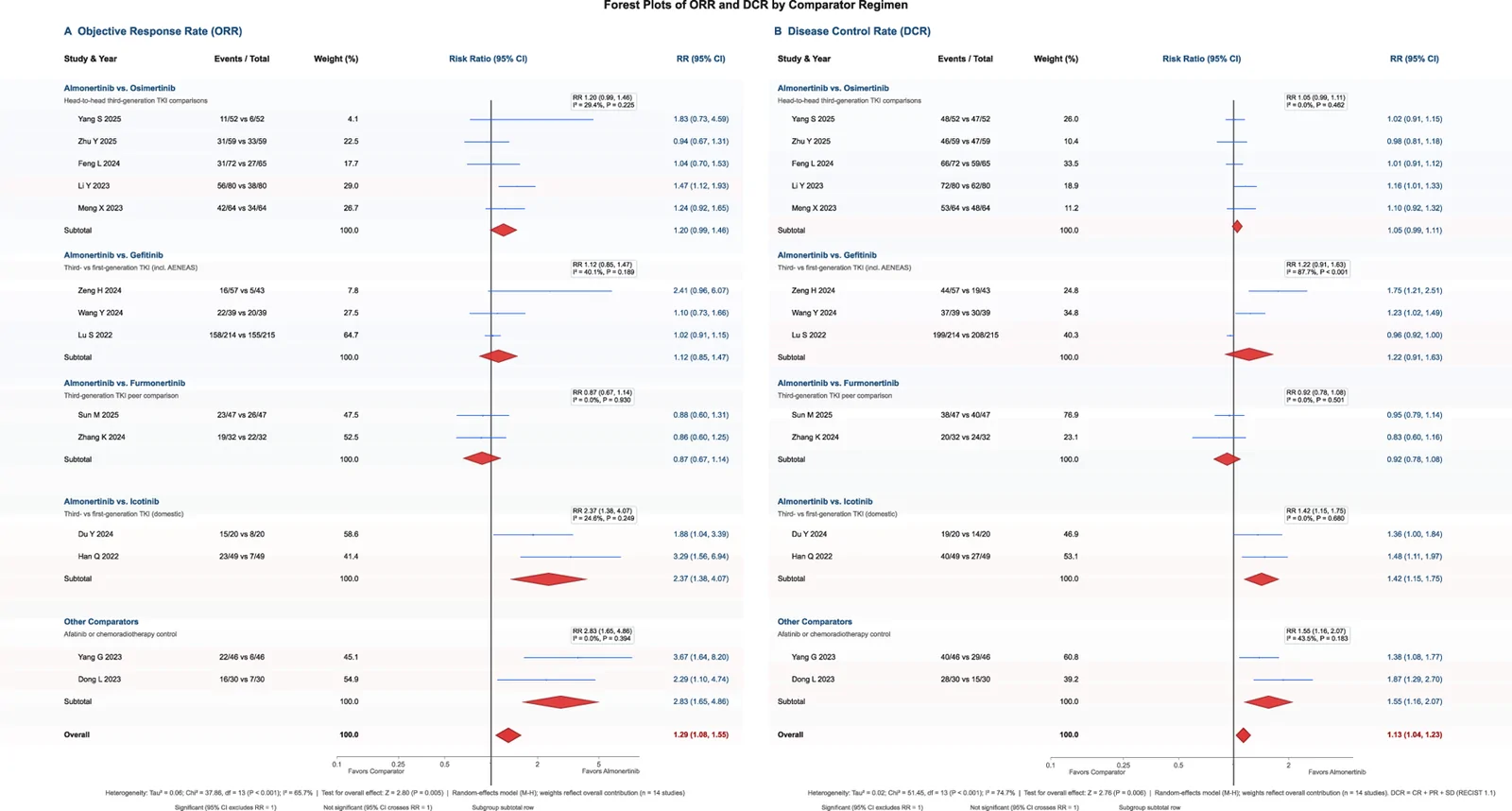
Subgroup forest plots of tumor response outcomes according to comparator regimen **(A)** Objective response rate was analyzed across 14 comparative studies stratified by comparator treatment, including osimertinib, gefitinib, furmonertinib, icotinib, and other comparator regimens. The comparison with osimertinib is clinically prioritized because head-to-head contrasts among third-generation EGFR-TKIs are central to treatment selection. The pooled ORR versus osimertinib did not reach statistical significance, whereas significant pooled effects were observed in the icotinib and other comparator subgroups; the gefitinib and furmonertinib subgroups also did not reach statistical significance. The overall pooled estimate favored almonertinib, but effect magnitude varied by comparator category **(B)** disease control rate was analyzed using the same comparator-based subgroup structure. Significant pooled effects were observed in the icotinib and other comparator subgroups, while the remaining subgroup estimates, including osimertinib, crossed the null value. The overall pooled estimate favored almonertinib, although subgroup estimates varied in precision and heterogeneity. These comparator-stratified analyses were intended to support clinically meaningful interpretation of the pooled results across distinct control regimens. Abbreviations: AENEAS, phase III randomized trial of almonertinib versus gefitinib in EGFR mutated non small cell lung cancer; CI, confidence interval; CR, complete response; DCR, disease control rate; df, degrees of freedom; EGFR, epidermal growth factor receptor; I2, inconsistency statistic estimating the percentage of total variation attributable to between study heterogeneity; M-H, Mantel Haenszel method; n, number of studies; ORR, objective response rate; P, probability value; PR, partial response; RECIST, Response Evaluation Criteria in Solid Tumors; RR, risk ratio; SD, stable disease; TKI, tyrosine kinase inhibitor.

### Safety profile across toxicity categories

Safety outcomes reported across the included comparative studies are summarized in Table 6, and pooled analyses according to toxicity category are presented in Supplementary Figure 2. Reported adverse events comprised grade 3 or higher adverse drug reactions, diarrhea, rash, nausea or vomiting, bone marrow toxicity, and hepatic or renal toxicity (Table 6). For grade 3 or higher adverse drug reactions, no significant difference was observed between the almonertinib and comparator groups (RR = 0.89, 95% CI 0.70 to 1.12; I^2^ = 10.0%) (Supplementary Figure 2A). Diarrhea was the only toxicity showing a significant reduction with almonertinib (RR = 0.72, 95% CI 0.55 to 0.92; I^2^ = 18.7%) (Supplementary Figure 2B). No significant differences were identified for rash (RR = 0.96, 95% CI 0.63 to 1.48; I^2^ = 53.5%) or nausea or vomiting (RR = 0.99, 95% CI 0.61 to 1.59; I^2^ = 40.5%), with pooled estimates remaining close to the null value (Supplementary Figures 2C,D). Bone marrow toxicity also showed no significant difference between treatment groups (RR = 1.10, 95% CI 0.73–1.65), although substantial between study heterogeneity was observed (I^2^ = 86.3%) (Supplementary Figure 2E). Similarly, hepatic or renal toxicity was comparable between groups (RR = 0.95, 95% CI 0.70 to 1.29; I^2^ = 39.9%) (Supplementary Figure 2F). Overall, the pooled safety analysis demonstrated a generally comparable toxicity profile between almonertinib and comparator regimens, with a significantly lower incidence of diarrhea observed in the almonertinib group. To enhance clinical interpretability of the safety findings, we additionally examined adverse events according to comparator type. Among studies contributing safety data, most used an EGFR-TKI control, whereas only one study used chemoradiotherapy without an EGFR-TKI as the comparator. Because a single chemoradiotherapy-controlled study cannot support a stable pooled chemotherapy subgroup, formal TKI-versus-chemotherapy stratified forest plots were not generated. Instead, we provide a narrative comparator-type appraisal. In TKI-controlled studies, the diarrhea advantage for almonertinib remained evident in a sensitivity synthesis excluding the chemoradiotherapy-controlled study (RR = 0.64, 95% CI 0.52–0.79). Bone marrow toxicity remained non-significant after the same restriction (RR = 1.14, 95% CI 0.58–2.24). In the single chemoradiotherapy-controlled study, diarrhea was not reduced with almonertinib (12/30 versus 9/30), underscoring that mixed-control safety estimates should not be generalized to chemotherapy-based comparators.

**TABLE 6:** Adverse outcomes of comparative studies of almonertinib.

Study	Year	Sample size (Intervention/Control)	Adverse drug reactions											
			Grade ≥3 ADR		Diarrhea		Rash		Nausea/vomiting		Bone marrow toxicity^‡^		Hepatic/renal toxicity^‡^	
			Intervention	Control	Intervention	Control	Intervention	Control	Intervention	Control	Intervention	Control	Intervention	Control
(Yao et al., 2025)	2025	79/121	—	—	23 (29.11%)	38 (31.40%)	26 (32.91%)	37 (30.58%)	—	—	33 (41.77%)^‡^	70 (57.85%)^‡^	—	—
(Yang et al., 2025)	2025	52/52	—	—	13 (25.00%)	23 (44.23%)	24 (46.15%)	12 (23.08%)	—	—	1 (1.92%)	1 (1.92%)	—	—
(Sun et al., 2025)	2025	47/47	—	—	2 (4.26%)	2 (4.26%)	4 (8.51%)	1 (2.13%)	5 (10.64%)	2 (4.26%)	4 (8.51%)^‡^	1 (2.13%)^‡^	1 (2.13%)	1 (2.13%)
(Zhu, 2025)	2025	59/59	—	—	3 (5.1%)	1 (1.7%)	2 (3.4%)	1 (1.7%)	—	—	—	—	—	—
(Feng et al., 2024)	2024	72/65	5 (6.94%)	7 (10.77%)	18 (25.00%)	23 (35.38%)	9 (12.50%)	12 (18.46%)	9 (12.50%)	11 (16.92%)	1 (1.39%)	1 (1.54%)	—	—
(Zhang et al., 2024)	2024	32/32	1 (3.13%)	0	2 (6.25%)	1 (3.13%)	4 (12.50%)	1 (3.13%)	—	—	4 (12.50%)^‡^	3 (9.38%)^‡^	3 (9.38%)	4 (12.50%)
(Zeng and Wang, 2024)	2024	57/43	12 (21.05%)	18 (41.86%)	—	—	—	—	—	—	—	—	—	—
(Wang and Wang, 2024)	2024	39/39	—	—	0	1 (2.56%)	0	3 (7.69%)	0	0	—	—	—	—
(Du, 2024)	2024	20/20	—	—	0	3 (15.00%)	1 (5.00%)	2 (10.00%)	—	—	—	—	—	—
(Li et al., 2023)	2023	80/80	12 (15.0%)	16 (20.0%)	8 (10.0%)	10 (12.5%)	4 (5.0%)	6 (7.5%)	8 (10.0%)	10 (12.5%)	8 (10.0%)^‡^	14 (17.5%)^‡^	32 (40.0%)^‡^	26 (32.5%)^‡^
(Meng et al., 2023)	2023	64/64	20 (31.25%)	19 (29.69%)	—	—	—	—	—	—	—	—	—	—
(Yang et al., 2023)	2023	46/46	—	—	1 (2.17%)	1 (2.17%)	1 (2.17%)	1 (2.17%)	1 (2.17%)^‡^	6 (13.04%)^‡^	—	—	—	—
(Dong, 2023)	2023	30/30	—	—	12 (40.00%)	9 (30.00%)	—	—	10 (33.33%)	8 (26.67%)	34 (113.33%)^‡^	31 (103.33%)^‡^	11 (36.67%)^‡^	8 (26.67%)^‡^
(Lu et al., 2022)	2022	214/215	78 (36.4%)	77 (35.8%)	35 (16.4%)	77 (35.8%)	50 (23.4%)	89 (41.4%)	49 (22.9%)^‡^	31 (14.4%)^‡^	141 (65.9%)^‡^	68 (31.6%)^‡^	154 (72.0%)^‡^	200 (93.0%)^‡^
(Han et al., 2022)	2022	49/49	—	—	0	1 (2.04%)	2 (4.08%)	3 (6.12%)	1 (2.04%)^‡^	6 (12.24%)^‡^	—	—	—	—

Intervention group = almonertinib or almonertinib-containing regimen; control group = comparator regimen; sorted by publication year, newest to oldest. Studies are listed by publication year (newest to oldest). Sample size is intervention group/control group. All values are reported as n (%) unless otherwise indicated. Grade ≥3 ADR, proportion of patients with any adverse drug reaction of grade ≥3 (CTCAE, or study-defined equivalent). When studies reported treatment-related grade ≥3 AEs (Li 2023) or grade ≥3 TEAEs (Lu 2022 AENEAS), this is stated in the column. Diarrhea, rash, and nausea/vomiting reflect study-reported incidence of each toxicity (any grade unless the source reported graded subsets only). “—” indicates not reported. ‡ Combined or derived values: Bone marrow toxicity = summed counts of leukopenia/white blood cell decrease, thrombocytopenia/platelet decrease, and anemia/hemoglobin decrease when reported separately; percentages use additive event counts divided by N (patients with more than one cytopenia may be counted more than once). Hepatic/renal toxicity = summed counts of reported hepatic dysfunction, transaminase elevation (ALT/AST), and renal dysfunction/creatinine increase. Lu 2022 (AENEAS): bone marrow = WBC, decrease + platelet decrease + anemia; hepatic/renal = ALT, increase + AST, increase + hepatic function abnormal + blood bilirubin increase. Li 2023: nausea/vomiting uses vomiting only (nausea not reported separately). Yang Guangxia 2023 and Han 2022: nausea/vomiting uses summed nausea and vomiting counts. Zhang 2024 grade ≥3 reflects creatine kinase elevation only (1/32 in almonertinib arm). Yao 2025 and Yang Shengxi 2025 did not report an overall grade ≥3 patient rate. Dong 2023 control received chemoradiotherapy without TKI., Feng 2024 is a non-parallel comparison (almonertinib second-line vs. osimertinib first-line). Zeng 2024 and Meng 2023 reported grade ≥3 toxicity only.

Abbreviations: ADR, adverse drug reaction; AE, adverse event; ALT, alanine aminotransferase; AST, aspartate aminotransferase; CPK, creatine phosphokinase; CTCAE, common terminology criteria for adverse events; N, number of patients; NV, nausea and vomiting; RCT, randomized controlled trial; TEAE, treatment-emergent adverse event.

### Sensitivity analysis of ORR and DCR

Sensitivity analyses are summarized in Supplementary Table 2, and the leave one out analyses for ORR and DCR are presented in Supplementary Figure 3. Sequential omission of individual studies did not materially alter the pooled estimates for either endpoint (Supplementary Table 2). For ORR, the pooled risk ratio after exclusion of each study ranged from 1.24 (95% CI 1.04–1.47) to 1.39 (95% CI 1.10–1.76), and all recalculated 95% confidence intervals remained above the null value (Supplementary Figure 3A). The corresponding heterogeneity varied modestly from 59.3% to 68.3%, without substantial changes from the overall estimate (Supplementary Table 2). A similar pattern was observed for DCR. After sequential omission of each study, pooled risk ratios ranged from 1.11 (95% CI 1.01–1.21) to 1.16 (95% CI 1.05–1.30), with all pooled estimates remaining statistically significant (Supplementary Figure 3B). Heterogeneity ranged from 66.6% to 76.7%, showing only limited variation across analyses (Supplementary Table 2). Overall, no individual study exerted a disproportionate influence on the pooled estimates for ORR or DCR, indicating that the primary efficacy results were robust to sequential study exclusion.

### Publication bias assessment

Publication bias was evaluated for ORR and DCR using Egger regression, Begg rank correlation, and the trim and fill method, with the corresponding results summarized in Supplementary Table 3 and visualized in Figure 7. Both efficacy endpoints were initially associated with statistically significant pooled effects before adjustment, including ORR (RR = 1.33, 95% CI 1.08–1.65) and DCR (RR = 1.14, 95% CI 1.03–1.26) (Supplementary Table 3). Egger regression and Begg rank correlation tests were statistically significant for both ORR (P = 0.0105 and P = 0.0248, respectively) and DCR (P = 0.0017 and P = 0.0186, respectively), indicating funnel plot asymmetry (Supplementary Table 3). The trim and fill procedure imputed five potentially missing studies for each endpoint (Supplementary Table 3). For ORR, the adjusted pooled estimate decreased from 1.33 (95% CI 1.08–1.65) to 1.08 (95% CI 0.82–1.43), with the confidence interval crossing the null value after adjustment (Figure 7A). Similarly, for DCR, the adjusted pooled RR decreased from 1.14 (95% CI 1.03–1.26) to 1.01 (95% CI 0.88–1.17), and statistical significance was no longer retained after imputation (Figure 7B). The funnel plots showed improved symmetry following imputation of the hypothetical missing studies, while the adjusted pooled estimates shifted toward the null value (Figure 7). Overall, the publication bias analyses indicated potential small study effects, and the trim and fill adjustment attenuated the pooled efficacy estimates for both ORR and DCR.

**FIGURE 7:**
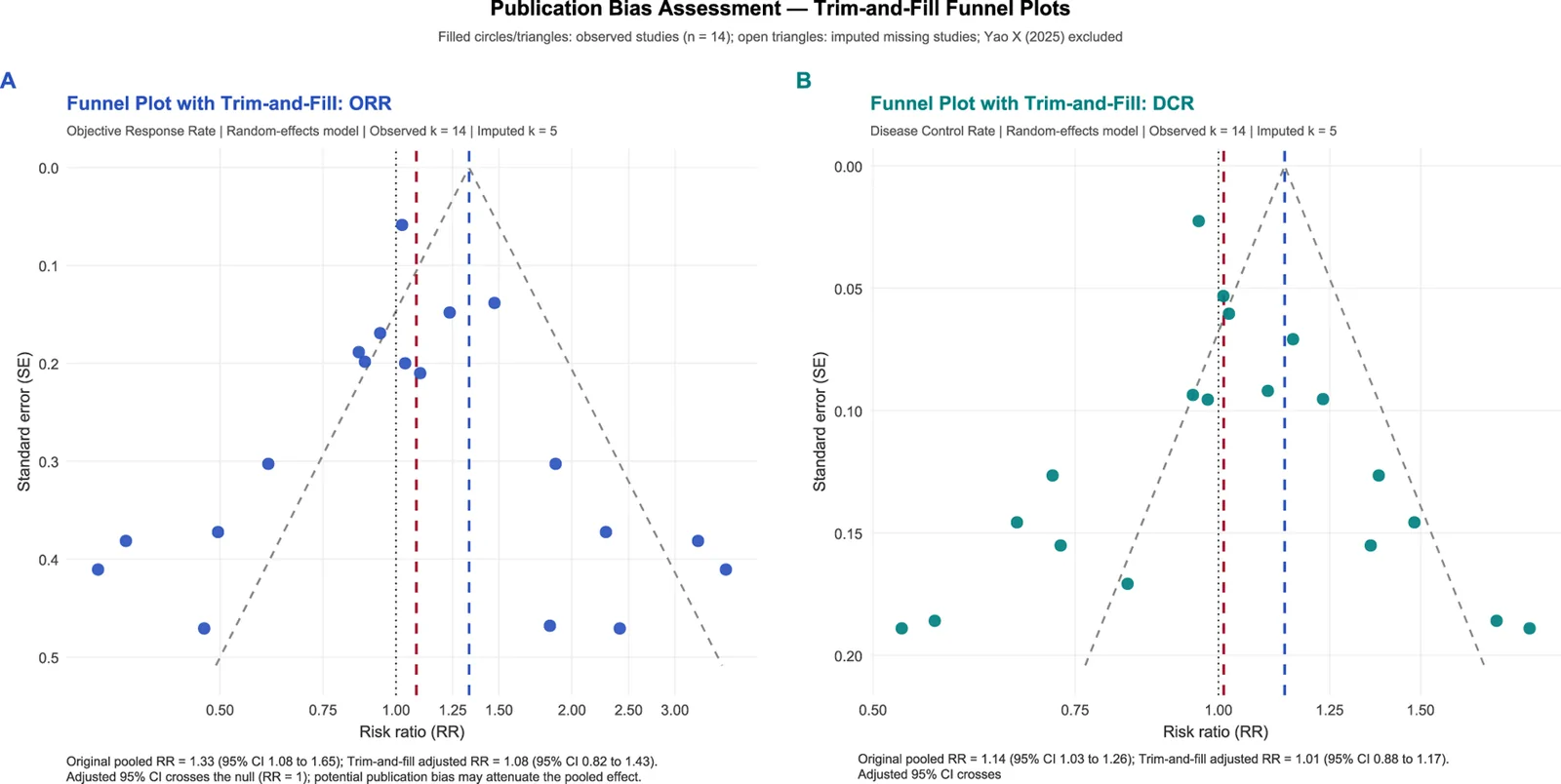
Publication bias assessment using trim and fill funnel plots for tumor response outcomes **(A)** Funnel plot with trim and fill analysis for objective response rate, showing 14 observed studies and 5 imputed studies. The original pooled estimate favored almonertinib, whereas the adjusted estimate shifted toward the null value after imputation, indicating that potential funnel plot asymmetry could attenuate the apparent ORR effect **(B)** funnel plot with trim and fill analysis for disease control rate, also showing 14 observed studies and 5 imputed studies. The adjusted pooled estimate similarly moved toward the null value, indicating that potential small study effects may influence the apparent DCR benefit. Filled symbols represent observed studies, open symbols represent imputed studies, the vertical reference line at RR = 1 represents the null effect, and adjusted estimates should be interpreted as sensitivity analyses rather than definitive correction for publication bias. Abbreviations: CI, confidence interval; CR, complete response; DCR, disease control rate; M H, Mantel Haenszel method; ORR, objective response rate; PD, progressive disease; PR, partial response; RECIST, Response Evaluation Criteria in Solid Tumors; RR, risk ratio; SD, stable disease; SE, standard error.

## Discussion

This systematic review and meta-analysis provides a timely synthesis of the comparative evidence on almonertinib-based therapy for EGFR-mutated NSCLC. By integrating 15 comparative studies involving 1,902 patients, our analysis suggests that almonertinib may provide favorable short-term tumor-control activity, with significantly higher ORR and DCR than comparator regimens. However, this apparent response advantage did not translate into a statistically significant pooled improvement in PFS or OS. Safety findings were generally reassuring: grade ≥3 adverse events, rash, nausea/vomiting, bone marrow toxicity, and hepatic/renal toxicity were broadly comparable between groups, while diarrhea appeared less frequent with almonertinib. Taken together, these results support almonertinib as a clinically relevant third-generation EGFR-TKI option, while also emphasizing that its comparative survival advantage remains incompletely established. Importantly, because control regimens were clinically heterogeneous, the overall response advantage should be read together with comparator-stratified findings (Figure 6), particularly the head-to-head comparison with osimertinib.

The improved ORR and DCR observed in this meta-analysis are biologically plausible. Almonertinib is an irreversible third-generation EGFR-TKI designed to inhibit both sensitizing EGFR mutations and T790M resistance mutation while relatively sparing wild-type EGFR. Early clinical data showed meaningful antitumor activity and manageable toxicity in T790M-positive advanced NSCLC (Yang et al., 2020). The phase III AENEAS trial further demonstrated superior PFS for aumolertinib over gefitinib in untreated EGFR-mutated locally advanced or metastatic NSCLC, establishing an important randomized foundation for its first-line use (Lu et al., 2022). Our pooled response results are directionally consistent with this clinical development pathway. Nevertheless, the lack of significant pooled PFS or OS benefit should be interpreted carefully rather than viewed as evidence of absence of benefit. Several factors may explain this discrepancy. First, survival endpoints were reported by fewer studies than response endpoints, and OS data were particularly immature or unavailable in many reports. Second, comparators were heterogeneous, including first-generation EGFR-TKIs, other third-generation EGFR-TKIs, chemotherapy-based regimens, and combined modality treatment. Third, some studies evaluated almonertinib in different treatment lines or combination contexts, which may have diluted survival estimates. Similar complexities have been observed in the broader EGFR-TKI field, where third-generation inhibitors may show clear PFS benefit while OS interpretation is affected by crossover, subsequent therapy, resistance mechanisms, and follow-up duration (Soria et al., 2018; Ramalingam et al., 2020).

Moderate-to-substantial heterogeneity was observed in the pooled analyses of ORR, DCR, and survival outcomes. Potential contributors include differences in comparator regimens (first-versus third-generation EGFR-TKIs, chemotherapy, or combined modalities), study design (randomized versus observational), treatment line and combination strategies, sample size, and follow-up duration. These sources of clinical and methodological diversity may explain why individual study estimates varied in magnitude, even when the overall direction of effect for tumor response generally favored almonertinib. Accordingly, the pooled estimates should be interpreted as indicating a consistent directional signal rather than a uniform effect size across all settings. Consistent with this interpretation, comparator-stratified analyses (Figure 6) showed that effect estimates varied by control regimen; the comparison with osimertinib did not demonstrate a statistically significant ORR or DCR advantage, indicating that pooled benefits against mixed controls should not be over-interpreted as superiority over other third-generation EGFR-TKIs.

The safety profile is one of the most clinically meaningful findings of this review. Almonertinib did not increase severe toxicity compared with control regimens, and diarrhea was significantly reduced. This may be relevant because gastrointestinal and dermatologic toxicities can influence treatment adherence, dose intensity, and quality of life during long-term EGFR-TKI therapy. Although cross-trial comparisons must be cautious, third-generation EGFR-TKIs were partly developed to reduce wild-type EGFR-related toxicities compared with earlier-generation agents. The AURA3 and FLAURA programs with osimertinib demonstrated the value of selective EGFR inhibition in improving efficacy while maintaining acceptable tolerability (Mok et al., 2017; Soria et al., 2018). Our findings suggest that almonertinib may share this favorable therapeutic profile. Another important implication concerns intracranial disease. Although this meta-analysis did not pool CNS-specific endpoints, brain metastases are common in EGFR-mutated NSCLC and represent a major determinant of prognosis and treatment selection. Recent evidence from AENEAS-related CNS analyses suggests that aumolertinib may reduce CNS progression risk compared with gefitinib, supporting further investigation of intracranial efficacy in future comparative studies (Lu et al., 2024). Future meta-analyses should prespecify CNS-PFS, intracranial response, and baseline brain metastasis subgroups when sufficient data become available. Nonetheless, safety signals should be interpreted in a comparator-specific manner. Most safety comparisons were against other EGFR-TKIs, and only one included study used chemoradiotherapy without TKI as the control; therefore, the overall reduction in diarrhea mainly reflects TKI-controlled evidence and should not be extrapolated as a safety advantage over chemotherapy or chemoradiotherapy. From a clinical pharmacology perspective, the lower diarrhea incidence with almonertinib is consistent with its third-generation design: irreversible inhibition of mutant EGFR (including T790M) with relative wild-type EGFR sparing, thereby reducing wild-type EGFR-related gastrointestinal toxicity (Shirley and Keam, 2022). Structurally, aumolertinib is related to other pyrimidine-based third-generation TKIs but contains a distinctive cyclopropyl modification that has been linked to mutant-selective activity and a favorable tolerability profile (Lu et al., 2022). This structure-activity rationale may partly explain our pooled safety findings, although definitive head-to-head biochemical superiority over all other third-generation agents should not be over-inferred (Yang et al., 2020).

The credibility of our findings is strengthened by the inclusion of both international databases and Chinese literature sources, which is particularly important because almonertinib has been widely developed and used in China. However, several limitations warrant emphasis. Most included studies were single-center, open-label, or retrospective, and only one study was a large multicenter double-blind phase III trial. Allocation concealment, blinding, sample size estimation, and intention-to-treat analysis were incompletely reported in many studies. Heterogeneity was moderate to substantial for ORR, DCR, and survival outcomes. Moreover, publication bias analyses suggested possible small-study effects: after trim-and-fill adjustment, the pooled ORR and DCR advantages were attenuated and no longer statistically significant. Therefore, the response benefit should be considered suggestive rather than definitive. Additionally, all included studies were conducted in China. This geographic concentration is understandable because almonertinib was developed in China and has been used mainly in Chinese clinical practice to date; however, it may still limit the generalizability of our findings to non-Chinese or non–East Asian populations.

Clinically, these findings support almonertinib as a reasonable EGFR-targeted therapeutic option, especially where drug accessibility, tolerability, and local treatment experience favor its use. The current evidence may be most persuasive for short-term disease control and tolerability, while definitive conclusions regarding OS, optimal sequencing, and superiority over other third-generation EGFR-TKIs require further high-quality evidence. Future trials should use head-to-head randomized designs, standardized adverse-event reporting, longer follow-up, molecular subgroup analyses, and patient-centered endpoints. Combination strategies may also deserve evaluation, but any incremental efficacy must be balanced against added toxicity, as highlighted by evolving osimertinib-combination evidence (Planchard et al., 2023). In particular, the absence of a statistically significant advantage over osimertinib in Figure 6 supports a cautious, comparator-specific clinical interpretation.

## Conclusion

In conclusion, this meta-analysis suggests that almonertinib-based therapy may improve tumor response and disease control in patients with advanced EGFR-mutated NSCLC without increasing severe toxicity, and may reduce diarrhea compared with comparator regimens. However, survival advantages remain uncertain, and the apparent response benefit may be influenced by heterogeneity and small-study effects. Almonertinib should therefore be regarded as a promising and clinically useful third-generation EGFR-TKI for advanced EGFR-mutated NSCLC, while further robust comparative studies are needed to define its precise position in modern treatment.

## Data Availability

The original contributions presented in the study are included in the article/Supplementary Material, further inquiries can be directed to the corresponding author.

## Supplementary material

Supplementary Figure 1 Landscape evidence and intervention flow plot of the 15 comparative studies evaluating almonertinib **(A)** Sample size weight plot showing the number of patients allocated to the intervention and control groups in each study, with total study sample size displayed on the right; **(B)** intervention pipeline showing almonertinib based treatment strategies and corresponding comparator regimens, together with scheduled treatment duration or continuation until disease progression; **(C)** outcome coverage matrix summarizing the availability of efficacy endpoints, adverse event endpoints, and reporting rigor across studies, including objective response rate, disease control rate, diarrhea, rash, nausea, grade 3 or higher adverse drug reactions, and overall adverse drug reaction profile. Abbreviations: ADR, adverse drug reaction; chemo, chemotherapy; DCR, disease control rate; d, day; n, number of patients or studies; ORR, objective response rate; PP, pemetrexed plus cisplatin; qd, once daily; RT, radiotherapy; seq., sequential therapy; tid, three times daily; TKI, tyrosine kinase inhibitor.

Supplementary Figure 2 Forest plot of adverse events by toxicity category in comparative studies of almonertinib **(A)** Grade 3 or higher adverse drug reactions showed no significant difference between almonertinib and comparator regimens; **(B)** diarrhea was significantly less frequent with almonertinib; **(C)** rash showed no significant difference between treatment groups; **(D)** nausea or vomiting showed comparable risk between groups; **(E)** bone marrow toxicity showed no significant difference but had substantial between study heterogeneity; **(F)** hepatic or renal toxicity showed no significant difference between groups. Risk ratios less than 1 favor almonertinib, horizontal lines indicate 95% confidence intervals, and red diamonds represent pooled subgroup estimates. Abbreviations: ADR, adverse drug reaction; AE, adverse event; CI, confidence interval; CTCAE, Common Terminology Criteria for Adverse Events; I2, inconsistency statistic representing the percentage of total variability attributable to between study heterogeneity; M-H, Mantel Haenszel method; RR, risk ratio; TEAE, treatment emergent adverse event.

Supplementary Figure 3 Leave one out sensitivity analysis of objective response rate and disease control rate **(A)** Leave one out sensitivity analysis for objective response rate showing the pooled risk ratio after sequential exclusion of each individual study under the random effects model. The pooled estimates remained consistently above the null value, with all 95% confidence intervals excluding RR = 1, indicating that omission of any single study did not materially influence the overall effect estimate or its statistical significance. The orange dashed line represents the pooled estimate from the full meta analysis, and the grey dashed line indicates the null value; **(B)** Leave one out sensitivity analysis for disease control rate performed using the same sequential exclusion procedure. The pooled risk ratios remained stable after omission of each study, and all recalculated 95% confidence intervals continued to exclude the null value, demonstrating that the overall disease control benefit was robust and was not driven by any individual study. Abbreviations: CI, confidence interval; DCR, disease control rate; LOO, leave one out; M H, Mantel Haenszel; ORR, objective response rate; RE, random effects; RECIST, Response Evaluation Criteria in Solid Tumors; RR, risk ratio.

## Glossary

ADR: Adverse Drug Reaction
AE: Adverse Event
AENEAS: Almonertinib/Aumolertinib Versus Gefitinib Phase III Trial Name
CBM: Chinese Biomedical Literature Database
CI: Confidence Interval
CNKI: China National Knowledge Infrastructure
CR: Complete Response
CTCAE: Common Terminology Criteria for Adverse Events
DCR: Disease Control Rate; df, Degrees of Freedom
ECOG: Eastern Cooperative Oncology Group
EGFR: Epidermal Growth Factor Receptor
EGFR-TKI: Epidermal Growth Factor Receptor Tyrosine Kinase Inhibitor;
Embase: Excerpta Medica Database
FACT-L: Functional Assessment of Cancer Therapy–Lung
FAS: Full Analysis Set
GRADE: Grading of Recommendations Assessment, Development and Evaluation
HR: Hazard Ratio
ITT: Intention-To-Treat
IWRS: Interactive Web Response System
KM/K-M: Kaplan–Meier
KPS: Karnofsky Performance Status
M-H: Mantel–Haenszel
mOS: Median Overall Survival
mPFS: Median Progression-Free Survival
n/N: Number
NA: Not Applicable
NGS: Next-Generation Sequencing
NOS: Newcastle–Ottawa Scale
NSCLC: Non-Small Cell Lung Cancer
ORR: Objective Response Rate
OS: Overall Survival
PD: Progressive Disease
PFS: Progression-Free Survival
po: Per Os/Orally
PP: Pemetrexed Plus Cisplatin
PR: Partial Response
PRISMA: Preferred Reporting Items for Systematic Reviews and Meta-Analyses
PS: Performance Status
qd: Once Daily
QOL/QoL: Quality Of Life
RCT: Randomized Controlled Trial
RD: Risk Difference
RE: Random-Effects Model
RECIST: Response Evaluation Criteria In Solid Tumors
RoB: Risk Of Bias
RR: Risk Ratio
RT: Radiotherapy
Rx: Treatment/Prescription
SAS: Statistical Analysis System
SD: Stable Disease/Standard Deviation
SE: Standard Error
seq.: Sequential Therapy
SF-36: 36-Item Short Form Health Survey
Sig.: Statistical Significance
SPSS: Statistical Package For The Social Sciences
T790M: Threonine-To-Methionine Substitution At EGFR Codon 790
tid: Three Times Daily
TKI: Tyrosine Kinase Inhibitor
TNM: Tumor-Node-Metastasis
VIP: Chinese Science And Technology Journal Database
WoS: Web Of Science
**χ** ^**2**^: Chi-Square Test
τ: Kendall Rank Correlation Coefficient
