# Supplementary material for: Comparative effectiveness and safety of almonertinib in EGFR-mutated non-small cell lung cancer: an integrated evidence synthesis
Source: Front Pharmacol. 2026 Oct 9;17:1932051. doi: 10.3389/fphar.2026.1932051 (PMC13690667; doi:10.3389/fphar.2026.1932051)

Landscape Evidence & Intervention Flow Plot

Table 3 comparative studies of almonertinib (n = 15) | Red dashed lines separate adjacent study rows

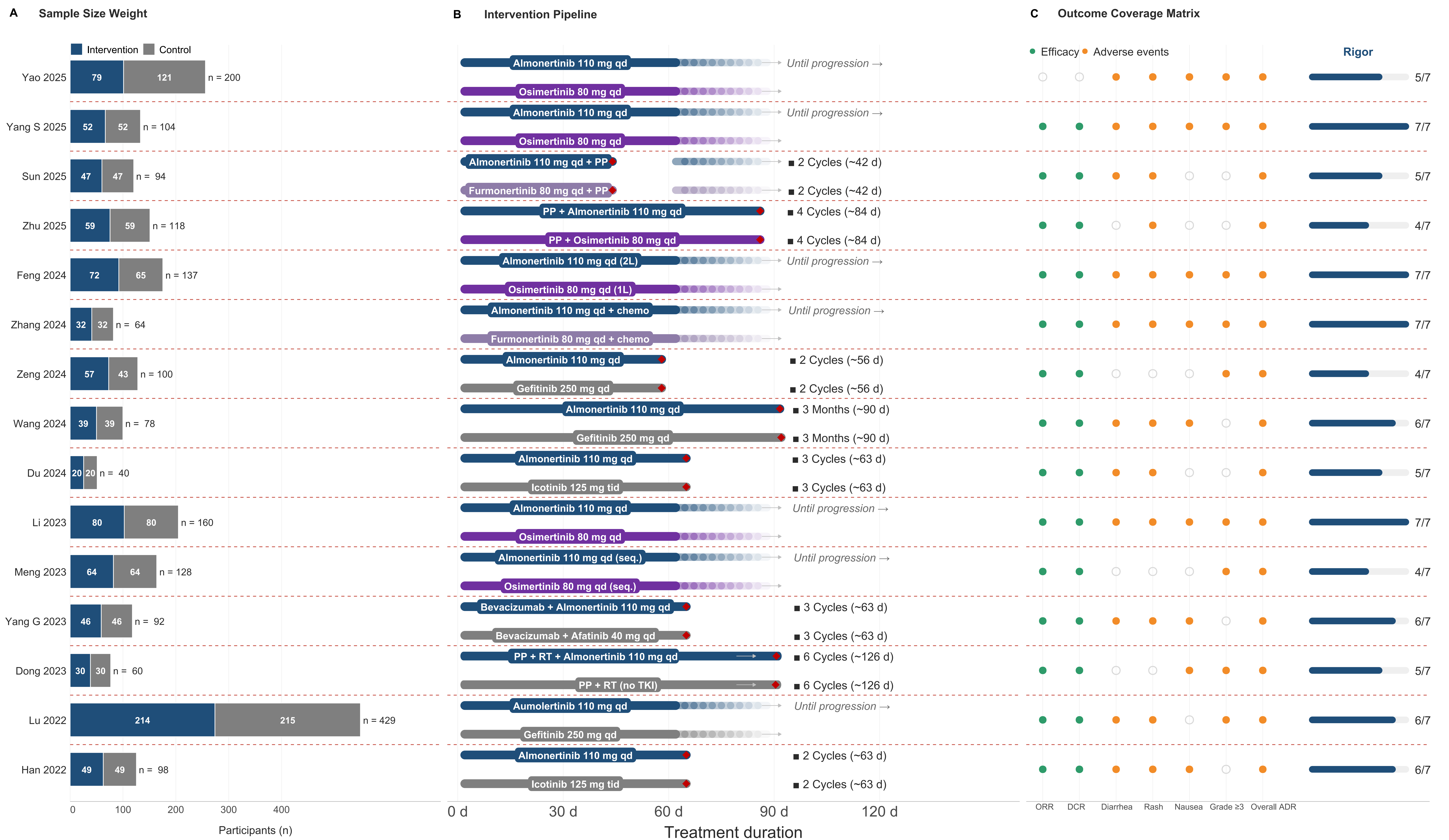

Supplement: Supplementary file 1 [file Supplementaryfile1.pdf]
