# Supplementary material for: Comparative effectiveness and safety of almonertinib in EGFR-mutated non-small cell lung cancer: an integrated evidence synthesis
Source: Front Pharmacol. 2026 Oct 9;17:1932051. doi: 10.3389/fphar.2026.1932051 (PMC13690667; doi:10.3389/fphar.2026.1932051)

Forest Plot of Adverse Events by Toxicity Category

Six toxicity subgroups | RR < 1 favors almonertinib (fewer events) | Blue CI lines; red subtotal diamonds

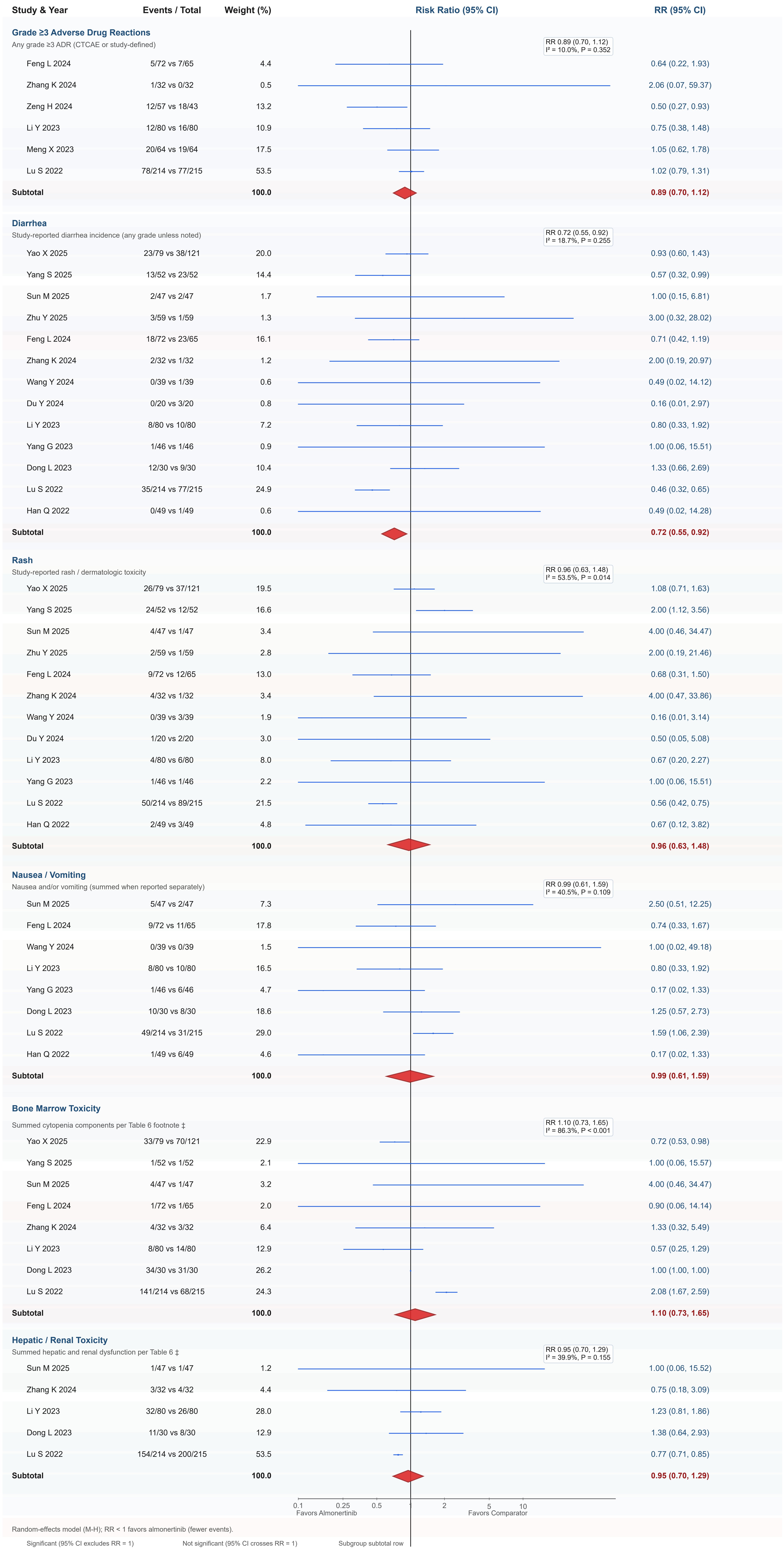

Supplement: Supplementary file 2 [file Supplementaryfile2.pdf]
