# Supplementary material for: Comparative effectiveness and safety of almonertinib in EGFR-mutated non-small cell lung cancer: an integrated evidence synthesis
Source: Front Pharmacol. 2026 Oct 9;17:1932051. doi: 10.3389/fphar.2026.1932051 (PMC13690667; doi:10.3389/fphar.2026.1932051)

Leave-One-Out Sensitivity Analysis

Each row shows the pooled RR after omitting one study; grey dashed = null (RR 1); orange dashed = full-model combined RR

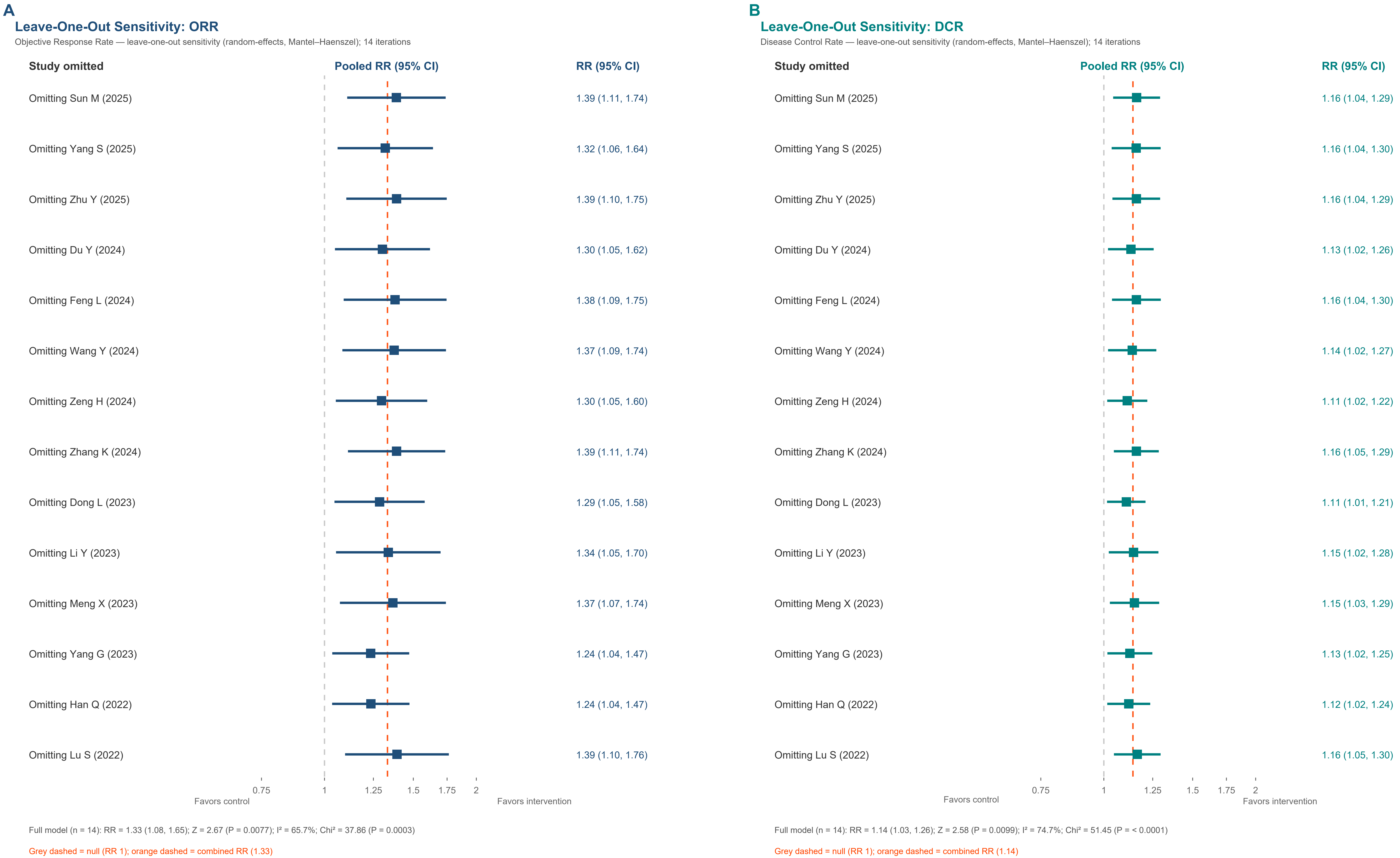

Supplement: Supplementary file 3 [file Supplementaryfile3.pdf]
