# Supplementary material for: Comparative effectiveness and safety of almonertinib in EGFR-mutated non-small cell lung cancer: an integrated evidence synthesis
Source: Front Pharmacol. 2026 Oct 9;17:1932051. doi: 10.3389/fphar.2026.1932051 (PMC13690667; doi:10.3389/fphar.2026.1932051)
Supplement: Supplementary file 4 [file Table1.docx]

**Supplementary Table 1. Integrated Analysis of ORR–DCR Relative Effect Concordance and Absolute Treatment Benefit Across Almonertinib Comparative Studies (n = 14)**

| **Study identification** | | **Sample & raw events** | **Relative efficacy — dual-endpoint ln(RR) framework** | | | | | | **Absolute efficacy — RD tiered ranking by ORR benefit** | | | | | **Integrated clinical inference** | | |
| --- | --- | --- | --- | --- | --- | --- | --- | --- | --- | --- | --- | --- | --- | --- | --- | --- |
| **Study** | **Year** | **N; ORR/DCR events (Rx/Ctrl)** | **ln(RR) ORR** | **ln(RR) DCR** | **Mean ln(RR)** | **Quadrant** | **RR ORR (95% CI)** | **P** | **RD ORR % (95% CI)** | **Rank by RDORR** | **RD DCR % (95% CI)** | **ΔRD %** | **Concordance** | **Relative-effect interpretation** | **Absolute-benefit interpretation** | **Synthesis** |
| Du Y [20] | 2024 | N=40 ORR 15/8 DCR 19/14 | 0.629 | 0.305 | 0.467 | I | 1.875 (1.036 to 3.394) | 0.038 | 35.000 (6.344 to 63.656) | 1 | 25.000 (2.760 to 47.240) | -10.000 | Concordant (+) | Both relative endpoints favor almonertinib | Both absolute benefits positive | Concordant benefit for almonertinib |
| Yang G [23] | 2023 | N=92 ORR 22/6 DCR 40/29 | 1.299 | 0.322 | 0.810 | I | 3.667 (1.639 to 8.200) | 0.002 | 34.783 (17.373 to 52.193) | 2 | 23.913 (6.904 to 40.922) | -10.870 | Concordant (+) | Both relative endpoints favor almonertinib | Both absolute benefits positive | Concordant benefit for almonertinib |
| Han Q [25] | 2022 | N=98 ORR 23/7 DCR 40/27 | 1.190 | 0.393 | 0.791 | I | 3.286 (1.556 to 6.940) | 0.002 | 32.653 (15.587 to 49.720) | 3 | 26.531 (8.881 to 44.180) | -6.122 | Concordant (+) | Both relative endpoints favor almonertinib | Both absolute benefits positive | Concordant benefit for almonertinib |
| Dong L [24]^#^ | 2023 | N=60 ORR 16/7 DCR 28/15 | 0.827 | 0.624 | 0.725 | I | 2.286 (1.102 to 4.743) | 0.026 | 30.000 (6.595 to 53.405) | 4 | 43.333 (23.338 to 63.329) | 13.333 | Concordant (+) | Both relative endpoints favor almonertinib | Both absolute benefits positive | Concordant benefit for almonertinib; DCR absolute gain exceeds ORR |
| Li Y [21] | 2023 | N=160 ORR 56/38 DCR 72/62 | 0.388 | 0.150 | 0.269 | I | 1.474 (1.123 to 1.933) | 0.005 | 22.500 (7.648 to 37.352) | 5 | 12.500 (1.233 to 23.767) | -10.000 | Concordant (+) | Both relative endpoints favor almonertinib | Both absolute benefits positive | Concordant benefit for almonertinib |
| Zeng H [18]^#^ | 2024 | N=100 ORR 16/5 DCR 44/19 | 0.881 | 0.558 | 0.720 | I | 2.414 (0.959 to 6.075) | 0.061 | 16.442 (1.346 to 31.538) | 6 | 33.007 (14.595 to 51.418) | 16.565 | Concordant (+) | Both relative endpoints favor almonertinib | Both absolute benefits positive | Concordant benefit for almonertinib; DCR absolute gain exceeds ORR |
| Meng X [22] | 2023 | N=128 ORR 42/34 DCR 53/48 | 0.211 | 0.099 | 0.155 | I | 1.235 (0.924 to 1.652) | 0.154 | 12.500 (-4.379 to 29.379) | 7 | 7.812 (-6.258 to 21.883) | -4.688 | Concordant (+) | Both relative endpoints favor almonertinib | Both absolute benefits positive | Concordant benefit for almonertinib |
| Yang S [13] | 2025 | N=104 ORR 11/6 DCR 48/47 | 0.606 | 0.021 | 0.314 | I | 1.833 (0.732 to 4.589) | 0.195 | 9.615 (-4.478 to 23.709) | 8 | 1.923 (-8.878 to 12.724) | -7.692 | Concordant (+) | Both relative endpoints favor almonertinib | Both absolute benefits positive | Concordant benefit for almonertinib |
| Wang Y [19]^#^ | 2024 | N=78 ORR 22/20 DCR 37/30 | 0.095 | 0.210 | 0.153 | I | 1.100 (0.729 to 1.661) | 0.650 | 5.128 (-16.969 to 27.226) | 9 | 17.949 (3.023 to 32.875) | 12.821 | Concordant (+) | Both relative endpoints favor almonertinib | Both absolute benefits positive | Concordant benefit for almonertinib; DCR absolute gain exceeds ORR |
| Lu S [8]^†§^ | 2022 | N=429 ORR 158/155 DCR 199/208 | 0.024 | -0.040 | -0.008 | IV | 1.024 (0.913 to 1.149) | 0.685 | 1.739 (-6.665 to 10.143) | 10 | -3.754 (-7.916 to 0.409) | -5.492 | Discordant | ORR favors almonertinib; DCR favors control | Absolute ORR and DCR benefits in opposite directions | Endpoint-specific divergence |
| Feng L [16] | 2024 | N=137 ORR 31/27 DCR 66/59 | 0.036 | 0.010 | 0.023 | I | 1.037 (0.700 to 1.534) | 0.858 | 1.517 (-15.046 to 18.080) | 11 | 0.897 (-8.604 to 10.399) | -0.620 | Concordant (+) | Both relative endpoints favor almonertinib | Both absolute benefits positive | Concordant benefit for almonertinib |
| Zhu Y [15] | 2025 | N=118 ORR 31/33 DCR 46/47 | -0.063 | -0.022 | -0.042 | III | 0.939 (0.674 to 1.309) | 0.712 | -3.390 (-21.358 to 14.578) | 12 | -1.695 (-16.438 to 13.048) | 1.695 | Concordant (−) | Both relative endpoints favor control | Both absolute benefits negative | Concordant benefit for control |
| Sun M [14] | 2025 | N=94 ORR 23/26 DCR 38/40 | -0.123 | -0.051 | -0.087 | III | 0.885 (0.600 to 1.305) | 0.537 | -6.383 (-26.539 to 13.773) | 13 | -4.255 (-19.426 to 10.915) | 2.128 | Concordant (−) | Both relative endpoints favor control | Both absolute benefits negative | Concordant benefit for control |
| Zhang K [17] | 2024 | N=64 ORR 19/22 DCR 20/24 | -0.147 | -0.182 | -0.164 | III | 0.864 (0.597 to 1.250) | 0.437 | -9.375 (-32.774 to 14.024) | 14 | -12.500 (-35.005 to 10.005) | -3.125 | Concordant (−) | Both relative endpoints favor control | Both absolute benefits negative | Concordant benefit for control |
| Pooled estimate (random-effects meta-analysis) | | | 0.258 | 0.123 | 0.191 | I | 1.33 (1.08 to 1.65) | 0.005 | Population-level concordant relative benefit: pooled ORR RR=1.295* and DCR RR=1.14* both favor almonertinib. Heterogeneity: I² ORR=65.661%, DCR=74.732%; P(Q) ORR=0.001, DCR=0.001. | | | | | | | |
| Cross-endpoint association (OLS regression) | | | ln(RR)DCR = 0.007 + 0.393 × ln(RR)ORR; r = 0.799; P = 0.001. Each 1.000-unit increase in ln(RR)ORR is associated with a 0.393 increase in ln(RR)DCR, indicating partial translation of objective response into disease control at the trial level. | | | | | | | | | | | | | |
| Overall scientific synthesis | | | Relative effects: 10/14 trials occupy Quadrant I (both ln(RR)ORR and ln(RR)DCR ≥ 0); 3/14 occupy Quadrant III. Pooled coordinates (ln(RR)ORR=0.258, ln(RR)DCR=0.123) confirm meta-analytic concordance favoring almonertinib. Absolute effects: 10/14 trials show positive RD for both endpoints; 3/14 show concordant negative RD. Rank-ordering by RDORR demonstrates steep early gains in objective response benefit with gradual attenuation across trials, while several mid-ranked studies retain larger DCR than ORR absolute gains (ΔRD > 0, #). Conclusion: almonertinib confers statistically significant pooled ORR and DCR advantages versus control, with meaningful cross-endpoint correlation and trial-level heterogeneity in absolute magnitude. | | | | | | | | | | | | | |

**Notes:** Relative-effect quadrant: Quadrant I — ln(RR)ORR ≥ 0 and ln(RR)DCR ≥ 0; Quadrant III — both < 0; Quadrants II and IV denote discordant relative directions between endpoints. Risk ratio (RR) and 95% CI were estimated with the Mantel–Haenszel approach; continuity correction (+0.5 to zero-event cells) was applied when needed. Risk difference (RD, %) = (event rate Rx − event rate control) × 100. Rank by RDORR assigns rank 1 to the study with the largest absolute ORR benefit, descending sequentially. Pooled estimates used random-effects meta-analysis (DerSimonian–Laird). Values are rounded to three decimal places; P values below 0.001 are reported as 0.001. † Lu S contributed the largest total sample (N = 429). § Discordant relative-effect quadrant (II or IV): ORR and DCR ln(RR) point in opposite directions. # ΔRD (DCR − ORR) ≥ 10.000 percentage points: absolute disease-control benefit substantially exceeds objective-response benefit. * Pooled ORR and DCR RR estimates: 95% CI excludes 1.000 (two-sided P < 0.05). **Abbreviations:** CI, confidence interval; DCR, disease control rate (CR + PR + SD); ΔRD, difference in risk difference (RD_DCR − RD_ORR, percentage points); ln(RR), natural logarithm of risk ratio; OLS, ordinary least squares linear regression; ORR, objective response rate (CR + PR); P, probability value; Q, Cochran Q statistic; RD, risk difference (absolute rate difference); RE, random effects; RECIST, Response Evaluation Criteria in Solid Tumors; RR, risk ratio; Rx, almonertinib (intervention) arm; τ², between-study variance; I², percentage of variability due to heterogeneity.
