# Supplementary material for: Comparative effectiveness and safety of almonertinib in EGFR-mutated non-small cell lung cancer: an integrated evidence synthesis
Source: Front Pharmacol. 2026 Oct 9;17:1932051. doi: 10.3389/fphar.2026.1932051 (PMC13690667; doi:10.3389/fphar.2026.1932051)
Supplement: Supplementary file 5 [file Table2.docx]

**Supplementary Table 2. Leave-one-out sensitivity analysis of objective response rate and disease control rate in comparative studies of almonertinib**

| No. | Study omitted (first author) | Year | Intervention (n) | Control (n) | ORR Intervention (events/total) | ORR Control (events/total) | DCR Intervention (events/total) | DCR Control (events/total) | RR (95% CI) | Sig. | I² | RR (95% CI) | Sig. | I² |
| --- | --- | --- | --- | --- | --- | --- | --- | --- | --- | --- | --- | --- | --- | --- |
|  |  |  | Almonertinib | Comparator | CR+PR | CR+PR | CR+PR+SD | CR+PR+SD | Random-effects | 95% CI excludes 1 | Heterogeneity | Random-effects | 95% CI excludes 1 | Heterogeneity |
| **—** | Overall (all 14 studies) | — | 861 | 841 | 485/861 | 394/841 | 750/861 | 669/841 | 1.33 (1.08, 1.65) | Yes | 65.7% | 1.14 (1.03, 1.26) | Yes | 74.7% |
| 1 | Yang S [13] | 2025 | 52 | 52 | 11/52 | 6/52 | 48/52 | 47/52 | 1.32 (1.06, 1.64) | Yes | 67.4% | 1.16 (1.04, 1.30) | Yes | 76.7% |
| 2 | Sun M [14] | 2025 | 47 | 47 | 23/47 | 26/47 | 38/47 | 40/47 | 1.39 (1.11, 1.74) | Yes | 67.0% | 1.16 (1.04, 1.29) | Yes | 76.4% |
| 3 | Zhu Y [15] | 2025 | 59 | 59 | 31/59 | 33/59 | 46/59 | 47/59 | 1.39 (1.10, 1.75) | Yes | 67.3% | 1.16 (1.04, 1.29) | Yes | 76.6% |
| 4 | Feng L [16] | 2024 | 72 | 65 | 31/72 | 27/65 | 66/72 | 59/65 | 1.38 (1.09, 1.75) | Yes | 68.2% | 1.16 (1.04, 1.30) | Yes | 76.7% |
| 5 | Zhang K [17] | 2024 | 32 | 32 | 19/32 | 22/32 | 20/32 | 24/32 | 1.39 (1.11, 1.74) | Yes | 66.5% | 1.16 (1.05, 1.29) | Yes | 76.0% |
| 6 | Zeng H [18] | 2024 | 57 | 43 | 16/57 | 5/43 | 44/57 | 19/43 | 1.30 (1.05, 1.60) | Yes | 65.9% | 1.11 (1.02, 1.22) | Yes | 72.1% |
| 7 | Wang Y [19] | 2024 | 39 | 39 | 22/39 | 20/39 | 37/39 | 30/39 | 1.37 (1.09, 1.74) | Yes | 68.3% | 1.14 (1.02, 1.27) | Yes | 74.6% |
| 8 | Du Y [20] | 2024 | 20 | 20 | 15/20 | 8/20 | 19/20 | 14/20 | 1.30 (1.05, 1.62) | Yes | 65.7% | 1.13 (1.02, 1.26) | Yes | 75.0% |
| 9 | Li Y [21] | 2023 | 80 | 80 | 56/80 | 38/80 | 72/80 | 62/80 | 1.34 (1.05, 1.70) | Yes | 64.3% | 1.15 (1.02, 1.28) | Yes | 74.9% |
| 10 | Meng X [22] | 2023 | 64 | 64 | 42/64 | 34/64 | 53/64 | 48/64 | 1.37 (1.07, 1.74) | Yes | 67.9% | 1.15 (1.03, 1.29) | Yes | 76.3% |
| 11 | Yang G [23] | 2023 | 46 | 46 | 22/46 | 6/46 | 40/46 | 29/46 | 1.24 (1.04, 1.47) | Yes | 59.3% | 1.13 (1.02, 1.25) | Yes | 73.7% |
| 12 | Dong L [24] | 2023 | 30 | 30 | 16/30 | 7/30 | 28/30 | 15/30 | 1.29 (1.05, 1.58) | Yes | 64.9% | 1.11 (1.01, 1.21) | Yes | 70.8% |
| 13 | Lu S [8] | 2022 | 214 | 215 | 158/214 | 155/215 | 199/214 | 208/215 | 1.39 (1.10, 1.76) | Yes | 63.3% | 1.16 (1.05, 1.30) | Yes | 66.6% |
| 14 | Han Q [25] | 2022 | 49 | 49 | 23/49 | 7/49 | 40/49 | 27/49 | 1.24 (1.04, 1.47) | Yes | 59.8% | 1.12 (1.02, 1.24) | Yes | 73.2% |

**Notes:** Studies are listed in descending order of publication year (2025 to 2022). Each data row reports the pooled random-effects meta-analysis after omitting the named study; the overall row includes all 14 eligible comparative studies. Yao X (2025) was excluded because objective response rate and disease control rate were not reported in the source publication (primary endpoint was progression-free survival). The intervention arm received almonertinib or an almonertinib-containing regimen; the control arm received the comparator regimen specified in each source study (third-generation EGFR-TKI, first/second-generation EGFR-TKI, or chemoradiotherapy in Dong L 2023). Tumor response was assessed per RECIST version 1.1. Objective response rate was defined as the proportion of patients with complete response (CR) or partial response (PR). Disease control rate was defined as the proportion with CR, PR, or stable disease (SD). Event counts for Wang Y (2024) were derived from reported PR and SD/PD data because CR was not reported separately (Table 5). Li Y (2023) progressive disease counts were derived when not explicitly tabulated. Risk ratios (RRs) and 95% confidence intervals (CIs) were calculated with the Mantel–Haenszel method under a random-effects (DerSimonian–Laird) model using R package meta (version 8.5). Continuity correction of 0.5 was applied only to studies with zero events in one arm (method = only 0). Leave-one-out sensitivity analysis was performed with the metainf function: each row shows the pooled estimate after exclusion of one study. Statistical significance (Sig.) was defined as a 95% CI that did not cross the null value of RR = 1. I² denotes the percentage of total variability due to between-study heterogeneity. Overall model statistics for ORR: χ² = 37.86, P = 0.0003, Z = 2.67, P = 0.0077; for DCR: χ² = 51.45, P = <0.0001, Z = 2.58, P = 0.0099. **Abbreviations:** ALM, almonertinib; CI, confidence interval; CR, complete response; DCR, disease control rate; EGFR, epidermal growth factor receptor; LOO, leave-one-out; MH, Mantel–Haenszel; ORR, objective response rate; PD, progressive disease; PR, partial response; RECIST, Response Evaluation Criteria in Solid Tumors; RR, risk ratio; SD, stable disease; Sig, statistical significance; TKI, tyrosine kinase inhibitor.
