# Supplementary material for: Comparative effectiveness and safety of almonertinib in EGFR-mutated non-small cell lung cancer: an integrated evidence synthesis
Source: Front Pharmacol. 2026 Oct 9;17:1932051. doi: 10.3389/fphar.2026.1932051 (PMC13690667; doi:10.3389/fphar.2026.1932051)
Supplement: Supplementary file 6 [file Table3.docx]

**Supplementary Table 3. Publication bias assessment for meta-analysis of objective response rate and disease control rate (Egger test, Begg test, and trim-and-fill method)**

| **Endpoint** | **Studies (k)** | **Pooled RR (95% CI)** | **Statistical significance†** | **Egger test Z statistic** | **Egger test P value** | **Begg test τ statistic** | **Begg test P value** | **Imputed studies (k)** | **Adjusted pooled RR (95% CI)** | **Significance after adjustment†** |
| --- | --- | --- | --- | --- | --- | --- | --- | --- | --- | --- |
|  | Included | M-H, random | CI excludes RR = 1 | Linear regression of ln(RR) on 1/SE |  | Rank correlation (Kendall τ) |  | Duval & Tweedie | Random-effects re-pooling | CI excludes RR = 1 |
| ORR | 14 | 1.33 (1.08, 1.65) | Yes | 3.027 | 0.0105 | 2.245 | 0.0248 | 5 | 1.08 (0.82, 1.43) | No |
| DCR | 14 | 1.14 (1.03, 1.26) | Yes | 4.018 | 0.0017 | 2.354 | 0.0186 | 5 | 1.01 (0.88, 1.17) | No |

**Notes:**Publication bias was evaluated for the 14 comparative studies with complete RECIST 1.1 response data in Table 5 (Yao X, 2025, excluded because objective response rate and disease control rate were not reported). Risk ratios (RRs) and 95% confidence intervals (CIs) were estimated with the Mantel–Haenszel method under a random-effects (DerSimonian–Laird) model using the R package meta (version 8.5). Continuity correction of 0.5 was applied only when an arm had zero events (method = only 0).Egger regression test: linear regression of the standard normal deviate (ln[RR]/SE) on precision (1/SE); a significant P value suggests funnel plot asymmetry and possible small-study/publication bias. Begg rank correlation test: Kendall τ rank correlation between the effect size and its variance; a significant P value likewise indicates potential asymmetry of the funnel plot. Trim-and-fill analysis (Duval and Tweedie): missing studies on the side of funnel plot asymmetry were imputed and the pooled RR was recalculated. Statistical significance (Sig.) was defined as a 95% CI that did not cross RR = 1. For both ORR and DCR, Egger and Begg tests were statistically significant (P < 0.05), and trim-and-fill imputed five studies for each endpoint. After adjustment, pooled RRs were attenuated and 95% CIs included the null value, suggesting that unpublished negative or null small studies could materially influence the apparent treatment effect. **Abbreviations:** CI, confidence interval; CR, complete response; DCR, disease control rate; M-H, Mantel–Haenszel; ORR, objective response rate; PD, progressive disease; PR, partial response; RECIST, Response Evaluation Criteria in Solid Tumors; RR, risk ratio; SD, stable disease; SE, standard error; Sig., statistical significance; τ, Kendall rank correlation coefficient.
